# Supplementary material for: Oxidation kinetics and non-Marcusian charge transfer in dimensionally confined semiconductors
Source: Nat Commun. 2023 Jul 10;14:4074. doi: 10.1038/s41467-023-39781-y (PMC10333350; doi:10.1038/s41467-023-39781-y)
Supplement: Supplementary file 1 — Supplementary Information [file 41467_2023_39781_MOESM1_ESM.pdf]

Supplementary Materials

Oxidation kinetics and non-Marcusian charge transfer in dimensionally confined semiconductors

Ning Xu[1,†], Li Shi[2,8,†], Xudong Pei[3,†], Weiyang Zhang[3], Jian Chen[1], Zheng Han[4], Paolo Samorì[5], Jinlan Wang[2,6,*], Peng Wang[7,*], Yi Shi[1,*], Songlin Li[1,*]

[1] School of Electronic Science and Engineering, National Laboratory of Solid-State Microstructures, and Collaborative Innovation Center of Advanced Microstructures, Nanjing University, Nanjing 210093, China

[2] Key Laboratory of Quantum Materials and Devices of Ministry of Education, Department of Physics, Southeast University, Nanjing 211189, China

[3] College of Engineering and Applied Sciences, Nanjing University, Nanjing 210023, China

[4] Institute of Opto-Electronics, Shanxi University, Taiyuan 030006, China

[5] University of Strasbourg, CNRS, ISIS UMR 7006, 8 allée Gaspard Monge, F-67000 Strasbourg, France

[6] Suzhou Laboratory, Suzhou 215125, China

[7] Department of Physics, University of Warwick, CV4 7AL Coventry, UK

[8] Present address: State Key Laboratory of Organic Electronics and Information Displays & Institute of Advanced Materials (IAM), Nanjing University of Posts and Telecommunications Nanjing 210023, China

[†] These authors contributed equally to this work.

*Corresponding authors. Emails: jlwang@seu.edu.cn, peng.wang.3@warwick.ac.uk, yshi@nju.edu.cn and sli@nju.edu.cn.

CONTENT

1. General challenges for quantifying oxidation behavior....................................- 3 -

2. Accumulation of experimental uncertainty ....- 3 -

3. Reliability of oxidation length extracted from PL imaging..............................- 4 -

4. Activation of basal oxidation mode..- 5 -

5. Preparation of top-view STEM samples ....- 6 -

6. Optical characterization of top-view STEM sample..- 8 -

7. Effect of electron bombardment on lattice vacancies ....- 8 -

8. STEM image filtering and lattice vacancy identification ....- 9 -

9. Raman characterization for excluding structural phase change ....- 19 -

10. Vacancy introduction revealed by XPS spectra ....- 21 -

11. Overview of extracting oxidation length for statistics ....- 23 -

12. Simulation on photothermal effect ....- 26 -

13. Service reliability due to degradation...- 26 -

14. Detecting activation threshold in photon energy..- 28 -

15. Control experiment in glovebox..- 28 -

16. Reaction rate versus substrate hydrophilicity ....- 29 -

17. Selection of humidity forms...- 30 -

18. UPS characterization ....- 30 -

19. Reaction paths and band diagram..- 33 -

20. Defect engineering via an alternative oxidant ....- 34 -

21. PL traced photooxidation for MoS_2...- 35 -

22. Statistics on [V] versus t_{pt} in MoS_2...- 37 -

1. General challenges for quantifying oxidation behavior

The grand challenge lies in the acquisition of excellent material platforms with well-defined parameter [V]. It is well known that atomic defects within a material represent a crucial crystallographic factor affecting its reaction reactivity. However, it is extremely challenging to obtain materials with well-defined [V] levels because of their non-uniform spatial distribution.

Conceptually speaking, any lattice defects are essential non-uniformity with respect to the perfect atoms staying in their lattice coordinates. In bulk materials, it is challenging or almost impossible to control the interior lattice defects in a “uniform” manner. Hence, it is impossible in bulk materials to carry out such kind of quantitative correlation of oxidation rate with [V] level, as did in 2D materials. In a word, 2D materials represent a unique platform to perform such an unprecedented study for fundamental reaction chemistry. In this sense, this work represents a really new effort for this fundamental issue.

On the other hand, although the atomic thickness and full expose of lattice atoms represents a structural advantage to control [V] levels on-demand, owing to the nature of randomness in vacancy creation, which follows the Poisson probability distribution, the distributions of [V] tend to be unconventionally broad (see Fig. 2k for the statistical data on [V] levels over 25 samples for each conditions). The intrinsic Poisson probability in spatial distribution (δ[V] ~ 20–30% in most cases) constitutes one of the primary origins of uncertainty in our experiment.

Moreover, during material degradation, defects tend to grow or spread along the sites or directions of raw defects, resulting in the behavior of clustering or aggregate of defects. Such a tendency of clustering represents another challenge to obtain data with low uncertainties, as required in most conventional experiments.

2. Accumulation of experimental uncertainty

As mentioned, the oxidation rate is a function of at least 4 fluctuated parameters (i.e., [V], RH, *T*, and *F*). The overall error in oxidation rate (*r*) is the result of the superposition of every parameter, that is, $\delta r = \delta[V] + \delta RH + \delta T + \delta F$. Given the fact that δ[V] ~20–30% (Fig. 2l), δRH ~5% (due to long-time drift), and δT ~5% (due to long-time drift) the accumulated error of δr may amount to 30–40% in most cases.

We have managed to minimize the experimental uncertainties by increasing the sampling numbers under each condition. In total, it took three years to prepare ca. 400 samples for this systematic/quantitative work covering 4 reaction parameters. To the best

of our knowledge, there is no relevant literature available regarding the quantitative survey of oxidation rate in either 2D or conventional bulk materials.

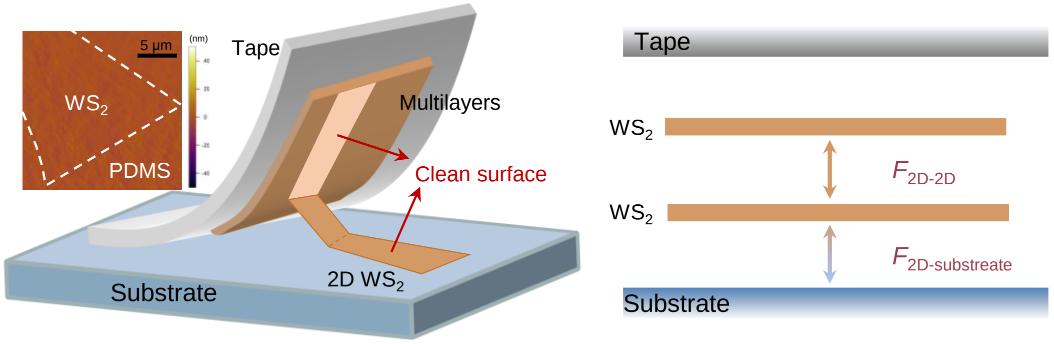

Supplementary Fig. 1 | Schematic diagram for mechanical exfoliation of TMDC flakes. The surfaces of TMDCs are formed in the last action of peeling off, and only the flakes with fresh and clean surfaces are used in the experiment. Inset: a AFM image for a typical WS_2 on PDMS substrate, indicating a clean surface after peeling.

3. Reliability of oxidation length extracted from PL imaging

The optical resolution of the PL imaging is about 200 nm when working under the 455 nm excitation. One may have doubts on its reliability and the accuracy of the experimental data. Hence, it would be wise to perform a complementary measurement for the estimation of oxidation length with other facilities such as line profile from AFM.

We clarify that the accuracy of PL imaging is comparable to AFM profiling in extracting the oxidation length, because it is a differential operation based on serial optical images and the relative low resolution in optics is removed during the differential operation. In Supplementary Fig. 2, we cross-checked the possible difference of length measured between PL and AFM measurements. In fact, the oxidation length determined by PL an AFM is 1.79±0.20 and 1.80±0.32 µm, respectively. Given the fluctuation of oxidation edges, the oxidation length extracted by these two facilities is quite comparable, which proves the reliability of our PL strategy.

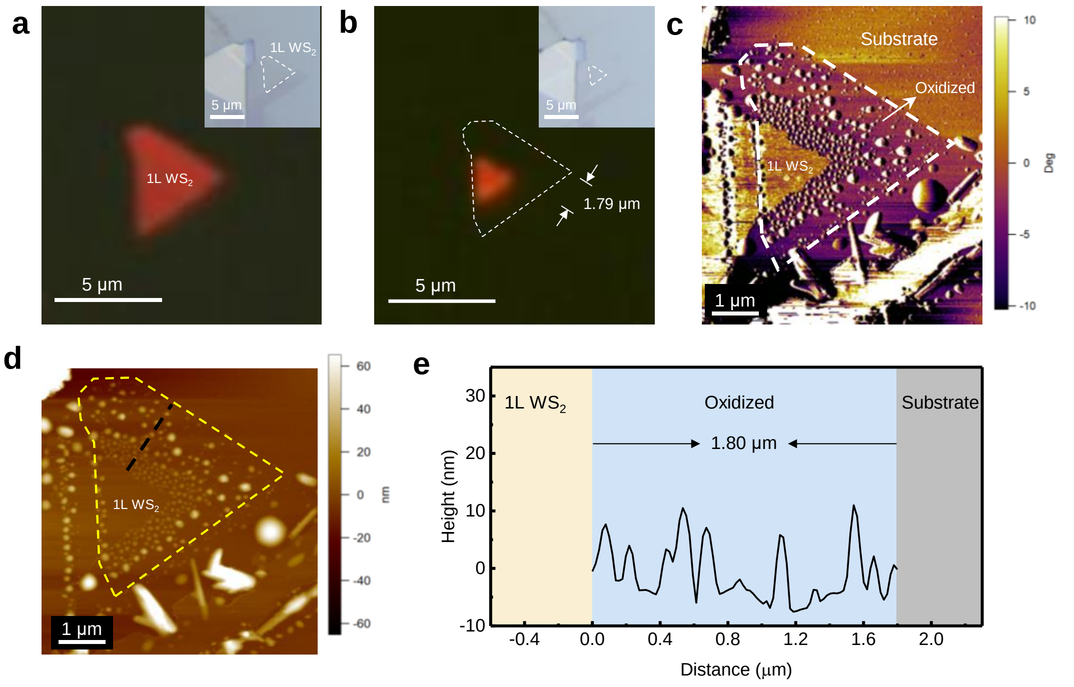

Supplementary Fig. 2 | Comparison of oxidation lengths measured between different methods from PL imaging and AFM. a,b, PL images for a partially oxidized 1L WS_2 before and after photo-oxidation. The insets show corresponding optical images. The oxidation length extracted from PL images is 1.79 μm. **c**, Corresponding AFM phase image. **d,** Surface morphology. **e**, Line profile from the dashed black line shown in **d**. The oxidation length recorded by AFM is 1.80 μm, which is close to that from PL imaging.

4. Activation of basal oxidation mode

Previous theoretical calculations revealed that the lattice atoms on the basal planes are more stable than those at the edges. Thus, the basal oxidation mode is normally deactivated. We found that the basal oxidation mode can be reproducibly activated in WS_2 (Supplementary Fig. 3), once the [V] level exceeds the threshold ~3×10^{14} cm^{-2} through appropriate engineering [V] levels at extended soaking time in H_2O_2 solutions or via long-time illumination (equivalent to increasing [V] levels).

A remarkable merit of PL imaging over reflection style is the high contrast in images of 2D materials. Due to the atomic thickness of 1L WS_2, it is difficult to discern the evolution of corrosion traces from a conventional microscope by optical reflection, as can be seen in the white-light reflection images before and after partial oxidation (Supplementary Fig. 3a,b). However, the situation is highly improved after adopting the PL imaging strategy in tracing the progressive oxidation process. The color labeling greatly enhance the image

contrast, so that even small features, such as the invisible narrow cracks across flakes in reflection mode, can be clearly discerned in the PL imaging. Such high brightness contrasts and spatial resolutions is favorable for increasing the experimental accuracy in estimating the oxidation rates.

Supplementary Fig. 3c,d shows two typical PL images taken before and after oxidation for a deeply defect engineered 1L WS_2. By comparing them, one can find that the cracks across the flake become wide and the triangular corrosion pits emerge from the lattice basal planes, which correspond to the trivial peripheral and activated basal oxidation modes, respectively. Interestingly, almost all the well-shaped corrosion triangles are aligned with the three edges are parallel to individual crystallographic directions (Supplementary Fig. 3e), implying the existence of oxidation anisotropy in the basal mode.

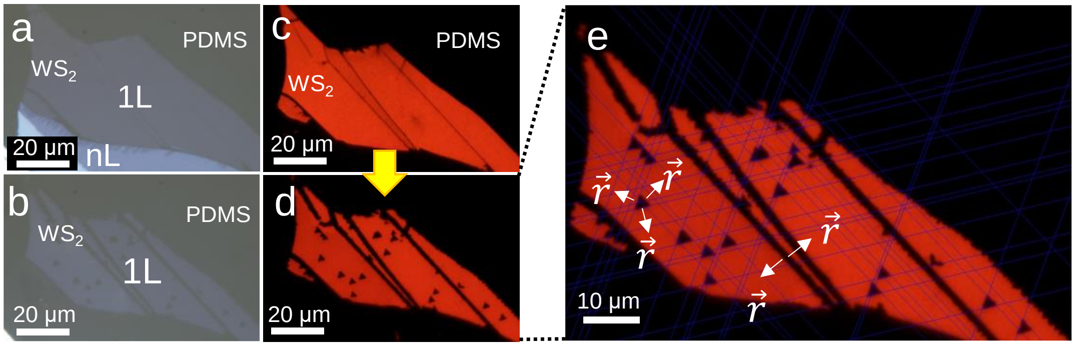

Supplementary Fig. 3 | Observation of basal oxidation mode in highly defective WS_2. **a**–**d**, Contrastive reflection and PL images recorded for a large-area 1L WS_2 flake before and after basal oxidation. In **d**, the randomly dispersed triangular corrosion pits emerge in the basal lattice planes, which is the characteristic of the basal oxidation mode. Note that the peripheral oxidation mode is always accompanied with the basal mode, since the activation energy of the former is lower than the latter. **e**, Enlarged PL image for **d**, in which the edges of the triangular corrosion pits are explicitly drawn and extended as guide lines for eyes, reflecting the three crystallographic directions in WS_2 as well as the oxidation anisotropy.

5. Preparation of top-view STEM samples

The top-view STEM samples were prepared with a polymer-assisted transfer method. At first, the monolayer WS_2 sheets initially exfoliated on PDMS substrate were transferred to SiO_2/Si substrates by using a home-made transfer facility (Supplementary Fig. 4a). In order to improve the adhesion between WS_2 sheets and SiO_2/Si substrates, the samples were then annealed at 120 °C for 2 min. Afterwards, PMMA polymers (used as the support for the atomically thin WS_2) were spin-coated on the substrates twice at 2000 rpm

(Supplementary Fig. 4b) and the $PMMA/WS_2/SiO_2/Si$ stacks were placed at room temperature for 5 hours to cure the PMMA support. In Supplementary Fig. 4c, a scotch tape with open window was used as auxiliary scaffold to peel off the $PMMA/WS_2$ bilayer by carefully sticking it to PMMA edges. Then the five-layered tape/$PMMA/WS_2/SiO_2/Si$ structure was immersed into a saturated sodium hydroxide (NaOH) solution for 5 min to detach the underlying SiO_2/Si substrate by etching the SiO_2 layer (Supplementary Fig. 4d). After that, the WS_2 sheets were aligned with and gently pressed onto a copper STEM grid that was placed in advance on SiO_2/Si substrate (Supplementary Fig. 4f). The scaffold of scotch tape was then removed leaving the $PMMA/WS_2$/STEM grid/SiO_2/Si stack baked at 140 ℃ for 10 min (Supplementary Fig. 4g), in which the PMMA support was softened to enhance the adhesion of WS_2 sheets to the holey carbon film of the STEM grid. Finally, the PMMA polymer was dissolved with acetone and the WS_2/STEM grid structure was achieved (Supplementary Fig. 4h).

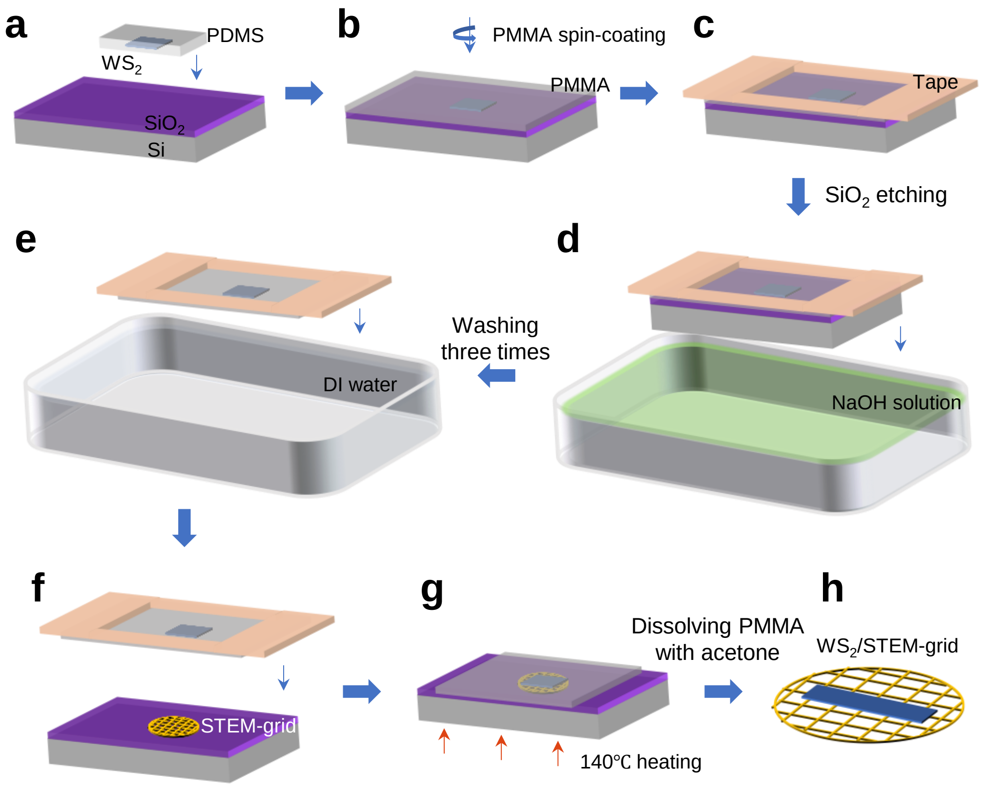

Supplementary Fig. 4 | Schematic illustration of wet transfer of WS_2 flakes onto STEM grids. a, Direct transfer of exfoliated WS_2 from PDMS substrates onto SiO_2/SiO_2 substrates; **b**, Spinning coating PMMA; **c**, Securing the top-edge of PMMA layer with perforated adhesive tapes; **d**, Etching underlying SiO_2 by saturated sodium hydroxide (NaOH) solution; **e**, Water rinse to remove NaOH residues; **f**, Transferring tape/$PMMA/WS_2$ stacks onto STEM grid; **g**, Removing tape from softening PMMA by heatup; **h**, Dissolving PMMA by acetone.

6. Optical characterization of top-view STEM sample

Supplementary Fig. 5 shows reflection and micro-zone PL images of WS_2 sheets supported by a STEM grid covered with holey carbon films. Supplementary Fig. 5a–c shows the reflection images from low to high magnification ratios. In Supplementary Fig. 5b, multiple WS_2 flakes with irregular shapes and a rectangular strip of metal Au can be seen, where the Au strip is used as a marker to facilitate the quick location of the WS_2 flakes. Supplementary Fig. 5c focuses on the 1L WS_2 flakes, which are denoted by dotted lines. Supplementary Fig. 5d shows the corresponding PL image of the 1L WS_2 area. The PL images are helpful to identify the real thickness of the WS_2 sheets on the STEM grids.

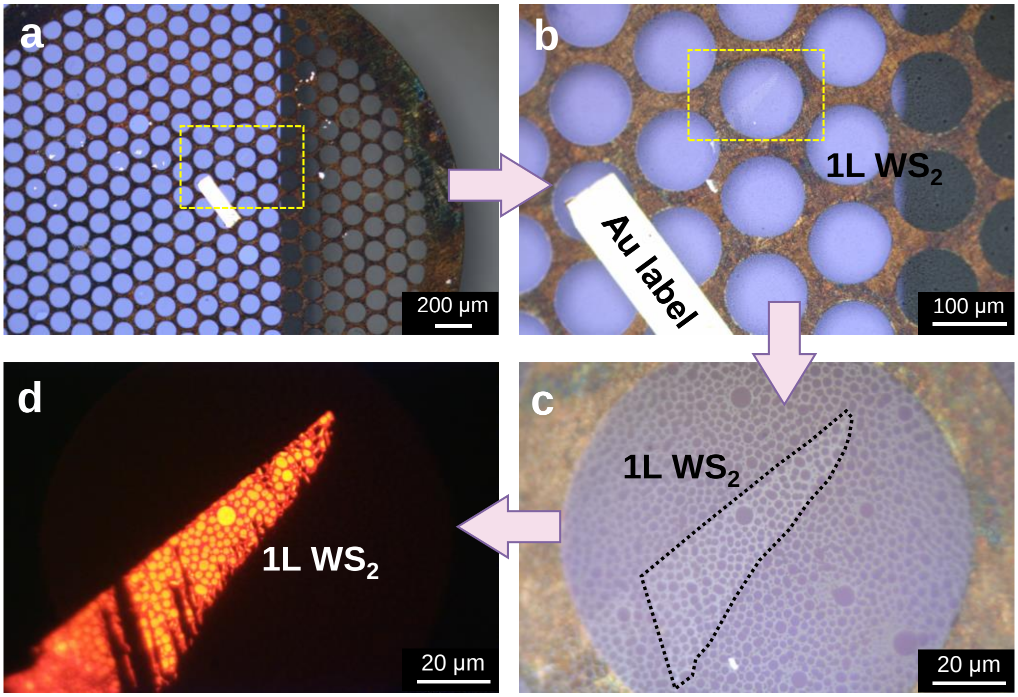

Supplementary Fig. 5 | Optical images for typical WS_2 flakes on STEM copper grids at various magnification ratios. a–c, Reflection images from low to high magnification ratios. The dashed yellow rectangles represent the areas to be enlarged in the next panels. The area surrounded by dashed black lines denotes the 1L WS_2, which can be clearly seen in the PL imaging mode. **d**, Corresponding PL image for the area shown in **c**.

7. Effect of electron bombardment on lattice vacancies

It is well known that the high-energy electron beams have strong bombardment effect on nano-materials. Such an adverse effect becomes even serious for the atomically thin WS_2 sheets. In order to shed light on it, we simply estimated the variation of numbers of sulfur vacancies in a local WS_2 area at different irradiation durations. Supplementary Fig.

6a–d shows the STEM images taken before and after 20, 40 and 80 s, respectively. Supplementary Fig. 6e summarizes the number of sulfur vacancies versus irradiation time where a monotonous trend was revealed between them. After 20-s irradiation, the number of vacancies slightly increases from 8 to 9, while it quickly increases to 14 after 40-s irradiation, amounting to a 75% inflation. Hence, the irradiation time was set below 10 sec for all imaging areas, to minimize the bombardment effect. As compared to the intrinsic vacancies present in pristine 1L WS_2, such a short exposure time brings extra errors below 10%.

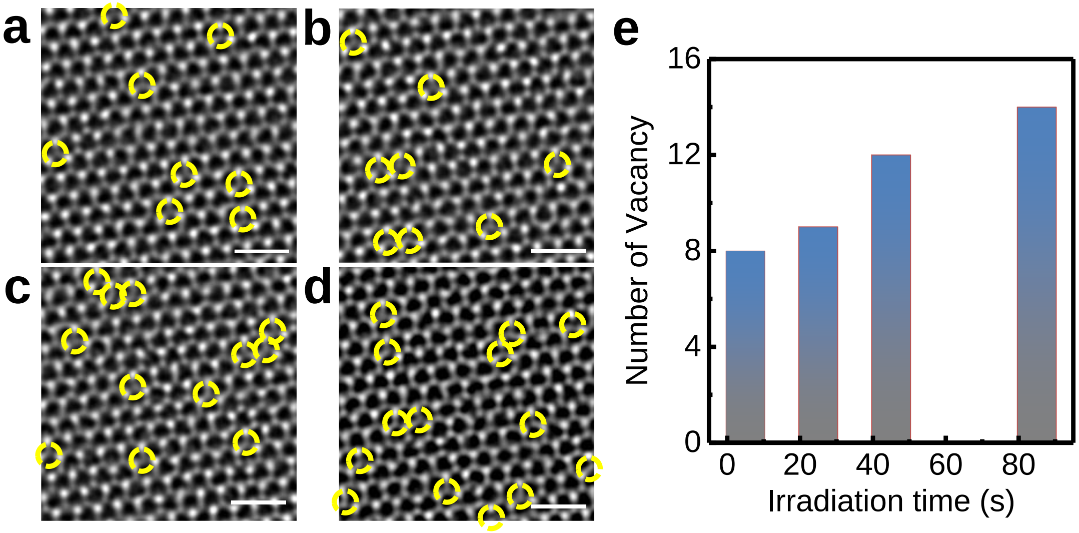

Supplementary Fig. 6 | Effect of electron bombardment in STEM on the number of lattice vacancy in a local region. a–d, ADF-STEM images for 1L WS_2 taken at different irradiation times. The probe current is 56 pA and electron irradiation time is 10 s for collecting an image. The observed sizes are all 4×4 nm^2. The dashed yellow circles denote the individual sulfur vacancies. **e**, Evolution of number of vacancies as irradiation time, where a roughly linear correlation is observed.

8. STEM image filtering and lattice vacancy identification

The spherical aberration-corrected scanning transmission electron microscopy (STEM) has been demonstrated to characterize the atomic vacancies in chalcogenide monolayers for its ultrahigh spatial resolution.[1,2] The structural characterization on our WS_2 samples was carried out with an aberration corrected Titan ChemiSTEM (FEI, USA). Supplementary Fig. 7a shows a typical raw image acquired with the STEM where polymer residues normally show up as areas with an enhanced brightness. Only the areas uncontaminated were selected for making statistics on the vacancy density. All raw images

were processed through the Wiener filtering and average background subtracting to increase the contrasts among various atoms and lattice vacancies.

Supplementary Fig. 7b,c shows typical atomically resolved STEM images before and after filtering. In the ADF imaging mode, heavy atoms would exhibit bright intensity in the greyscale images. Hence, the brightest dots and their adjacent slightly less bright ones correspond to the tungsten and sulfur atoms, respectively, and the darkest areas encircled by six tungsten and sulfur atoms can be ascribed to the centers of the hexagonal W-S atomic rings. The lattice vacancies, i.e., loss of atoms, would exhibit reduced brightness as compared to those of occupied sites, as indicated by the red arrows.

Alternatively, the atomic vacancies can be discerned by comparing the contrast profile lines. Supplementary Fig. 7d,e compares the profile lines covering a few W-S atomic pairs taken from the pristine and filtered images. It can be seen that the profile line becomes much smoother after noise filtering. Hence, the sulfur vacancy can be quickly counted by searching the sites with lower intensity. In this way, the vacancy density can also be accurately estimated.

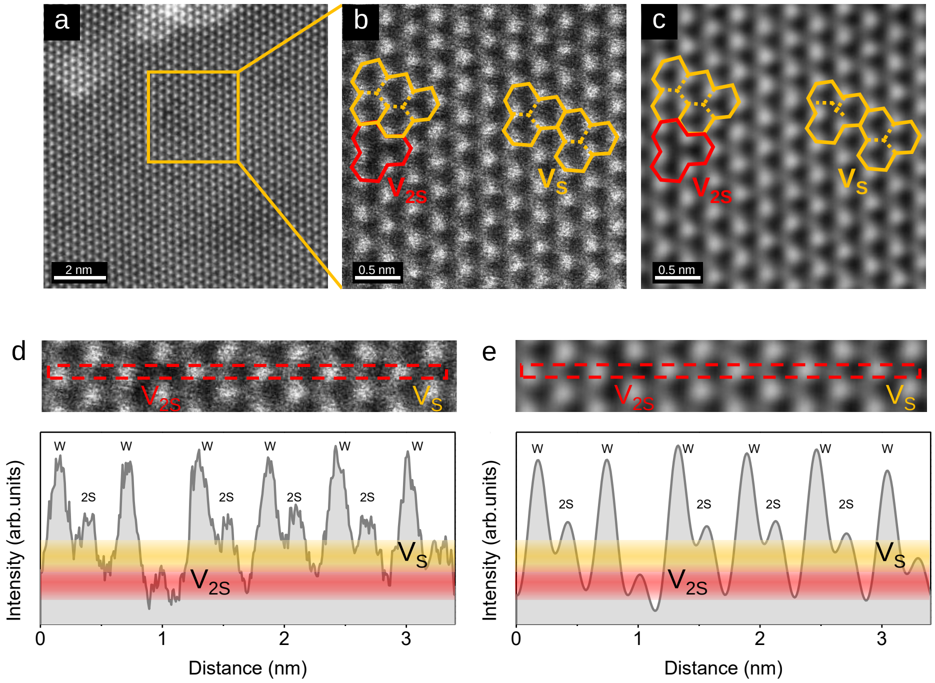

Supplementary Fig. 7 | Coexistence of double and single sulfur vacancies in defect-engineered WS_2. a, A typical raw STEM image for a 25-min treated 1L WS_2. **b** and **c**, Enlarged STEM image taken from **a** and corresponding filtered image containing double (V_{2S}) and single (V_S) sulfur vacancies. **d** and **e**, Line profiles taken from the local area of raw and filtered STEM images containing V_{2S} and V_S.

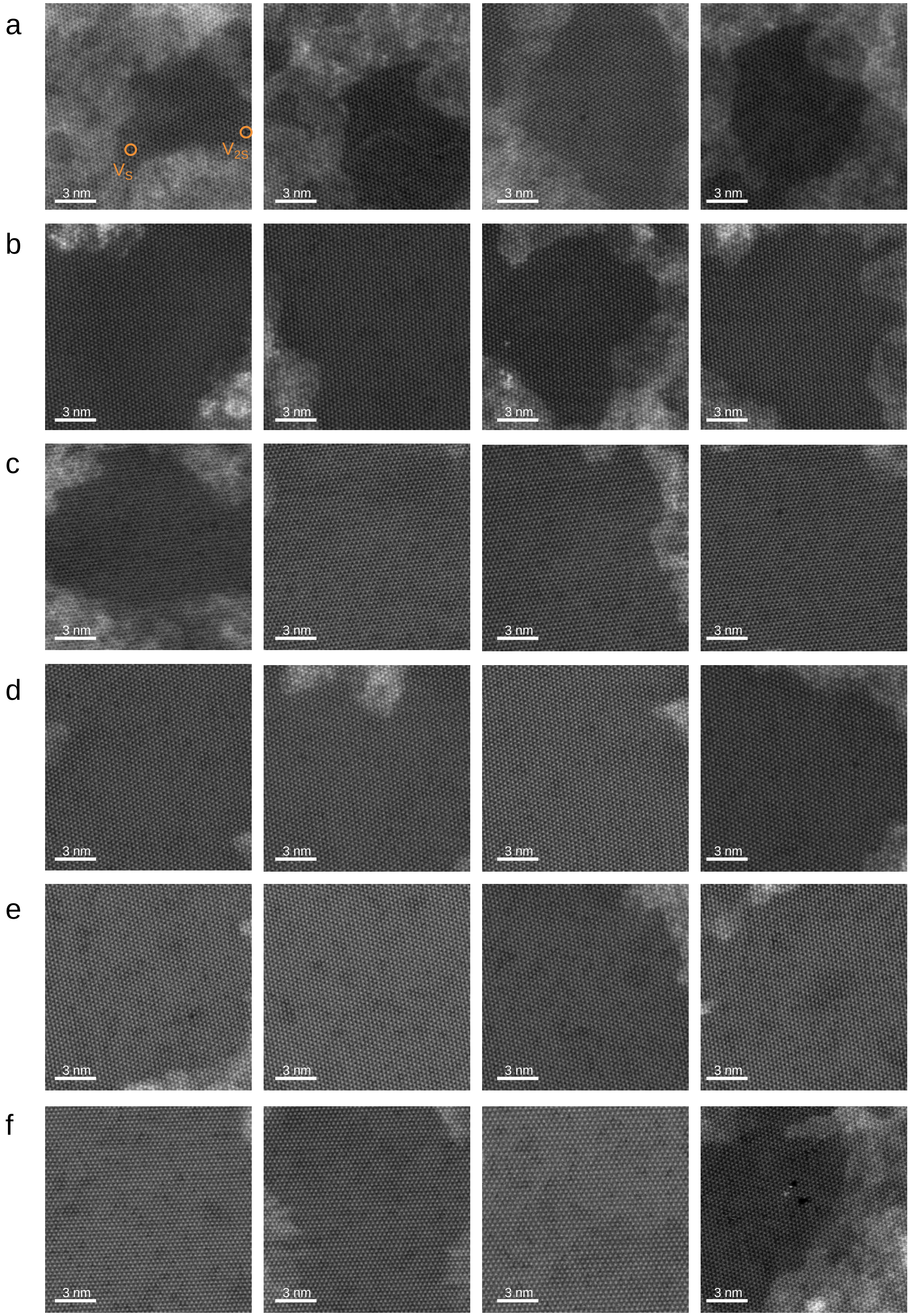

Supplementary Fig. 8 | Large-area (15×15 nm^2) raw STEM images for 1L WS_2 after treating with H_2O_2 solution for different durations: **a**, 0 min; **b**, 10 min; **c**, 15 min; **d**, 20 min; **e**, 25 min; **f**, 30 min.

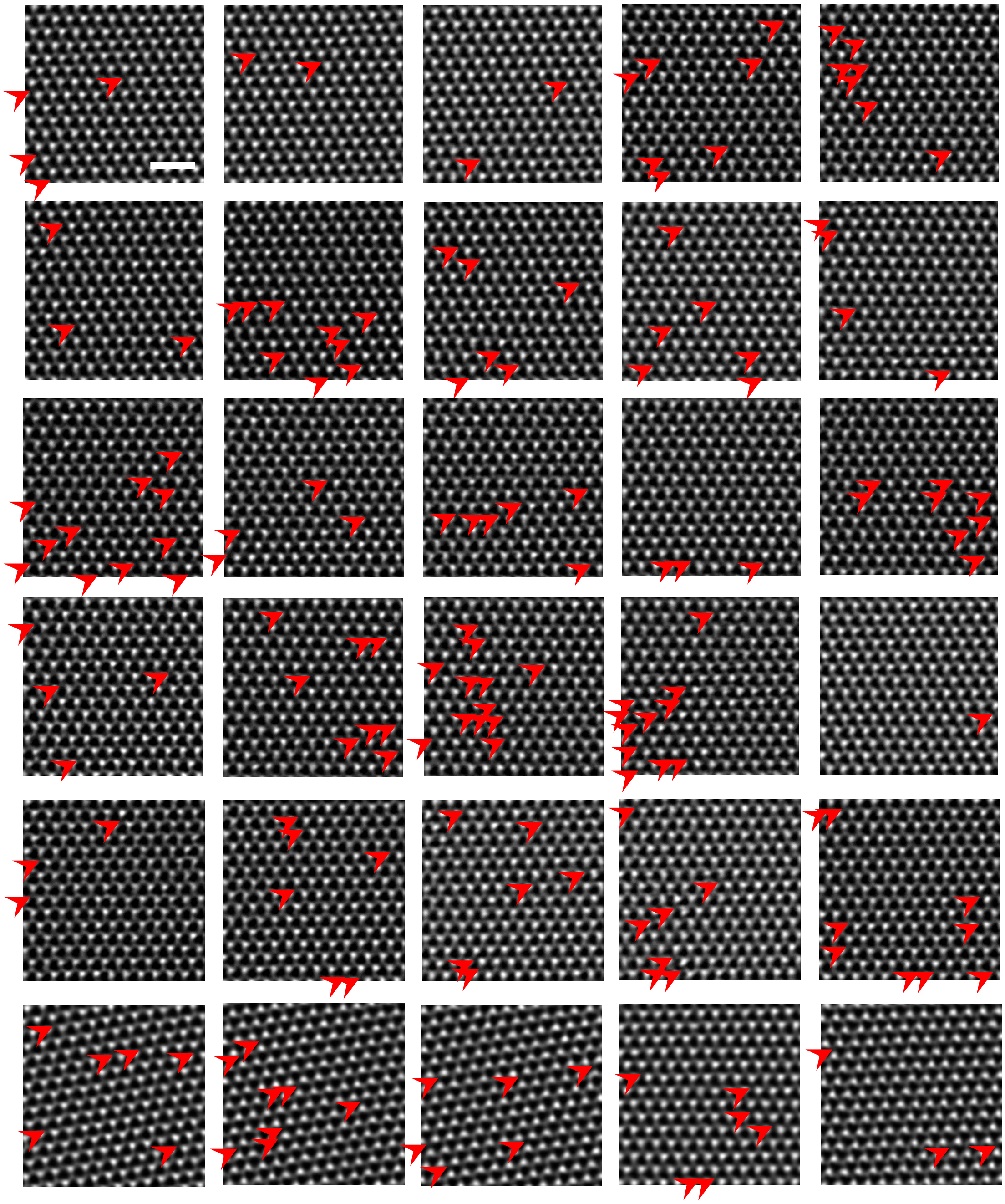

Supplementary Fig. 9 | Statistics of sulfur vacancy distribution in pristine monolayer WS_2 without pretreatment. Scale bar: 1 nm.

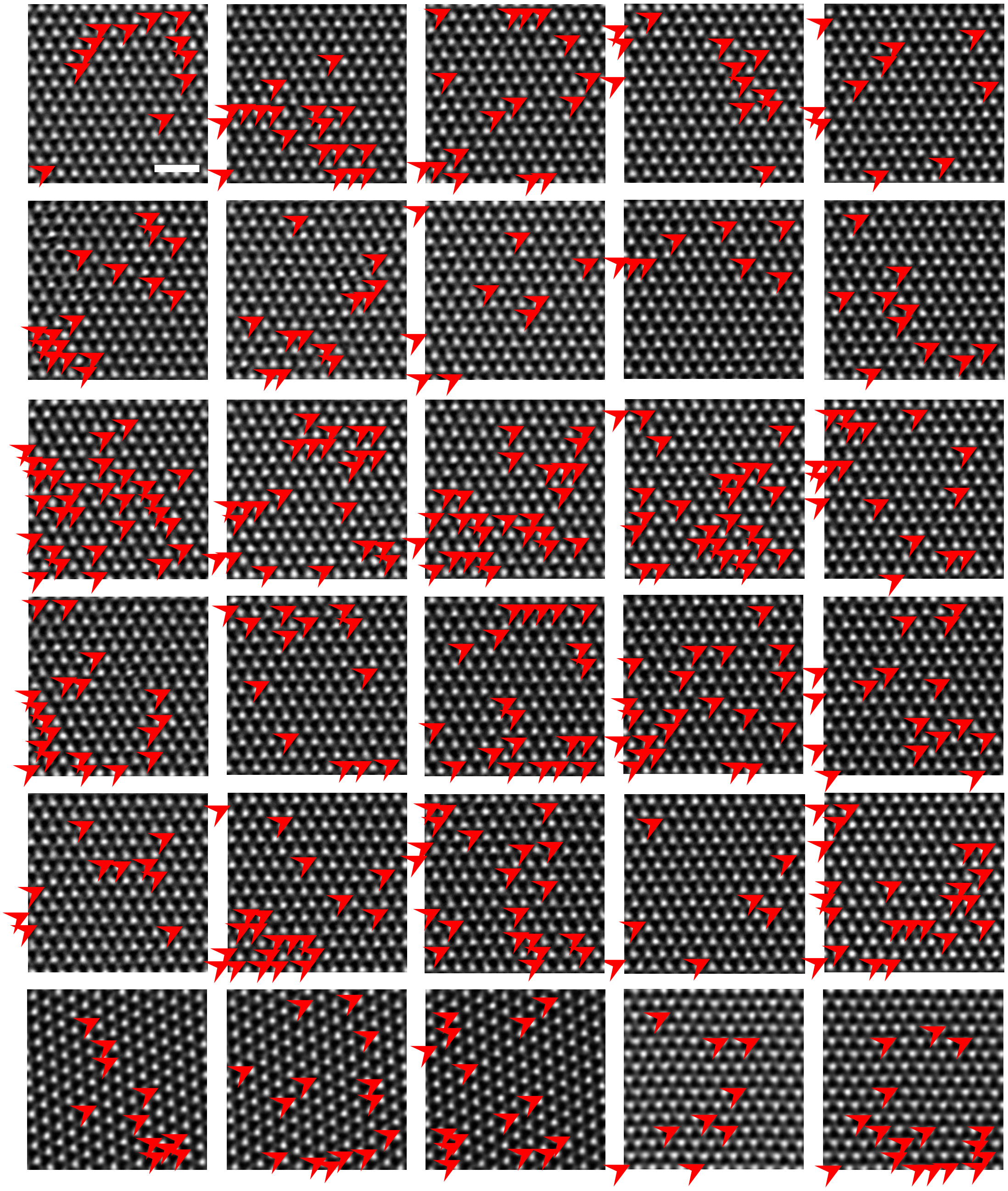

Supplementary Fig. 10 | Statistics of sulfur vacancy distribution in monolayer WS_2 after pretreatment in H_2O_2 solution for 10 min. Scale bar: 1 nm.

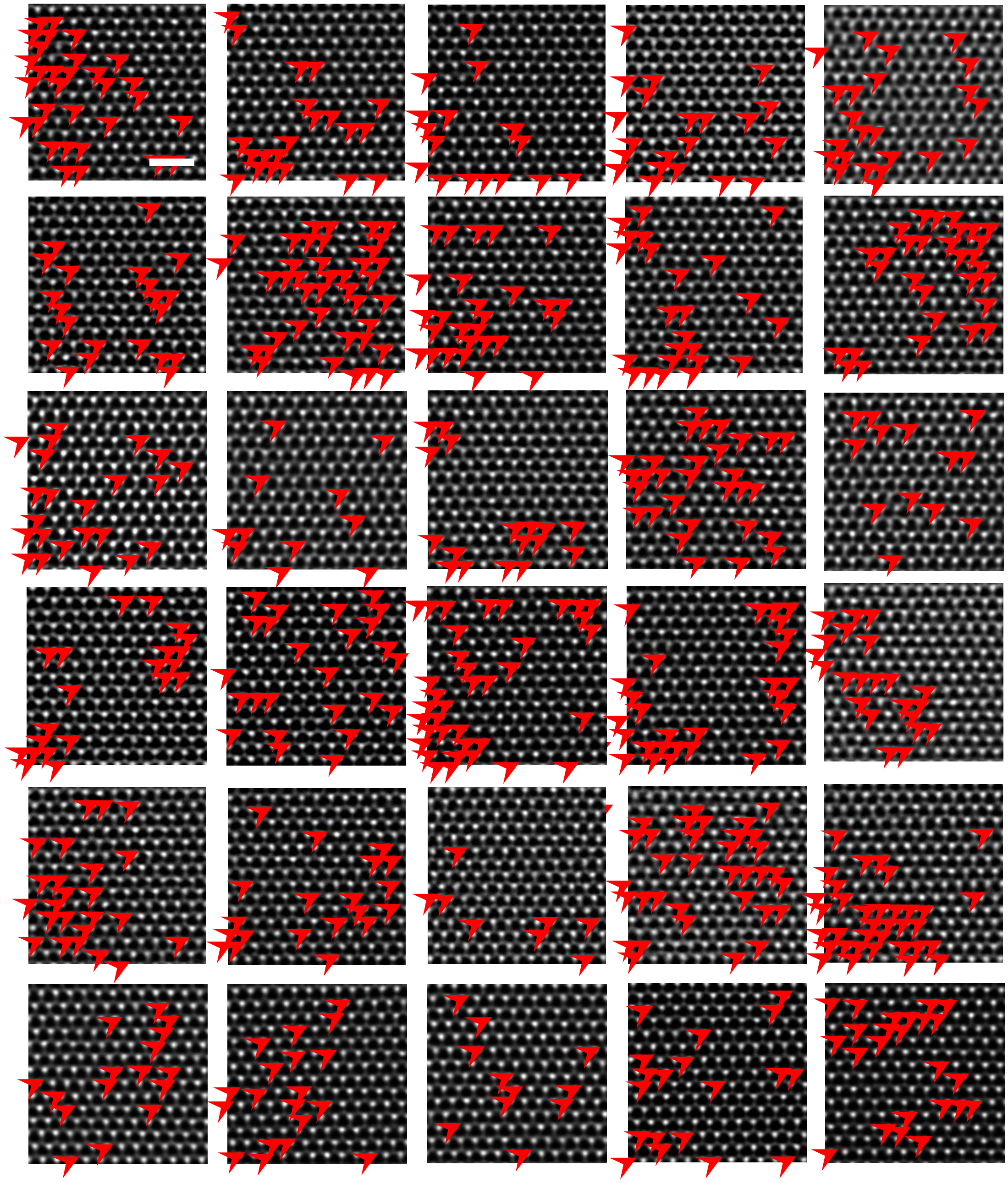

Supplementary Fig. 11 | Statistics of sulfur vacancy distribution in monolayer WS_2 after pretreatment in H_2O_2 solution for 15 min. Scale bar: 1 nm.

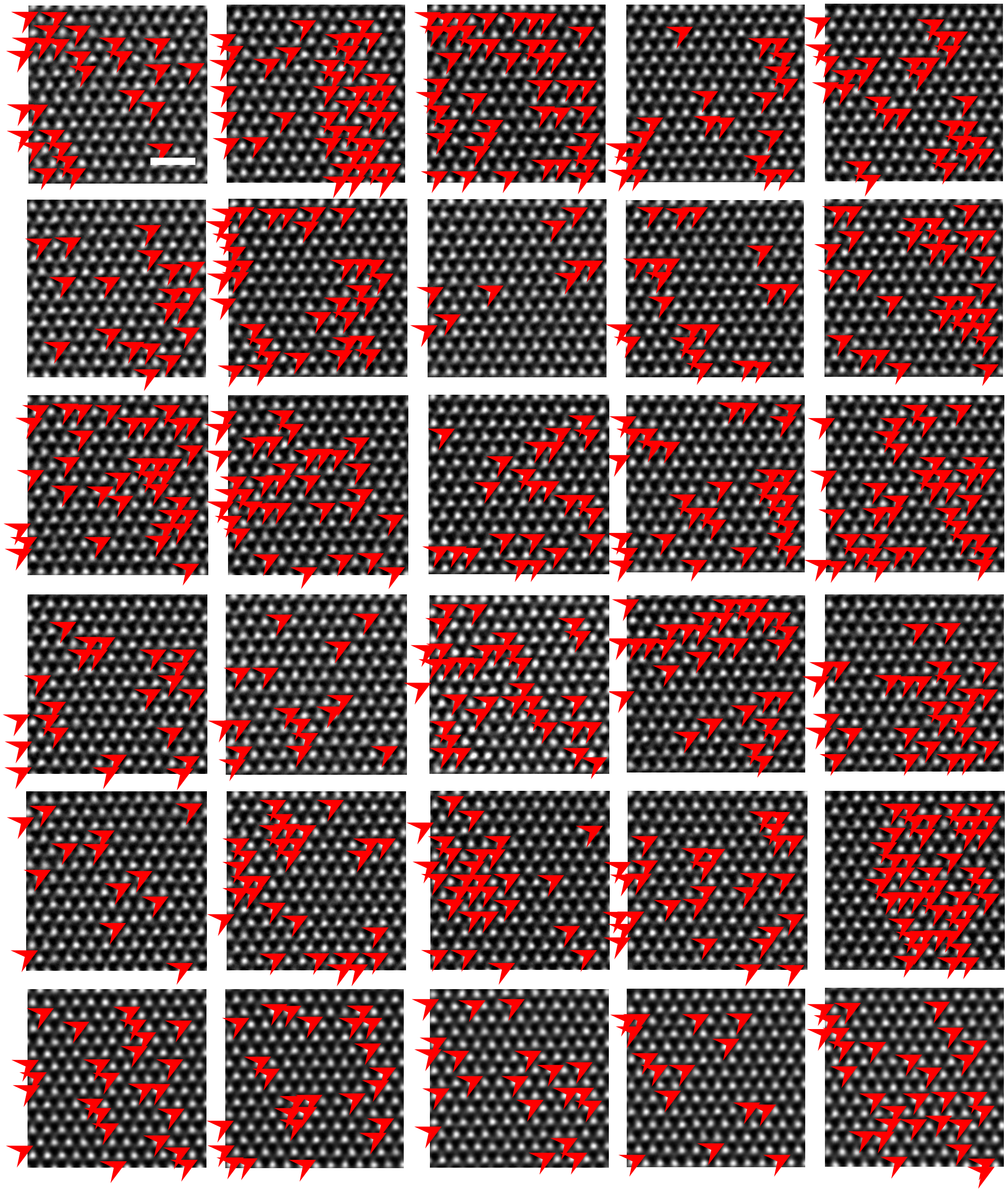

Supplementary Fig. 12 | Statistics of sulfur vacancy distribution in monolayer WS_2 after pretreatment in H_2O_2 solution for 20 min. Scale bar: 1 nm.

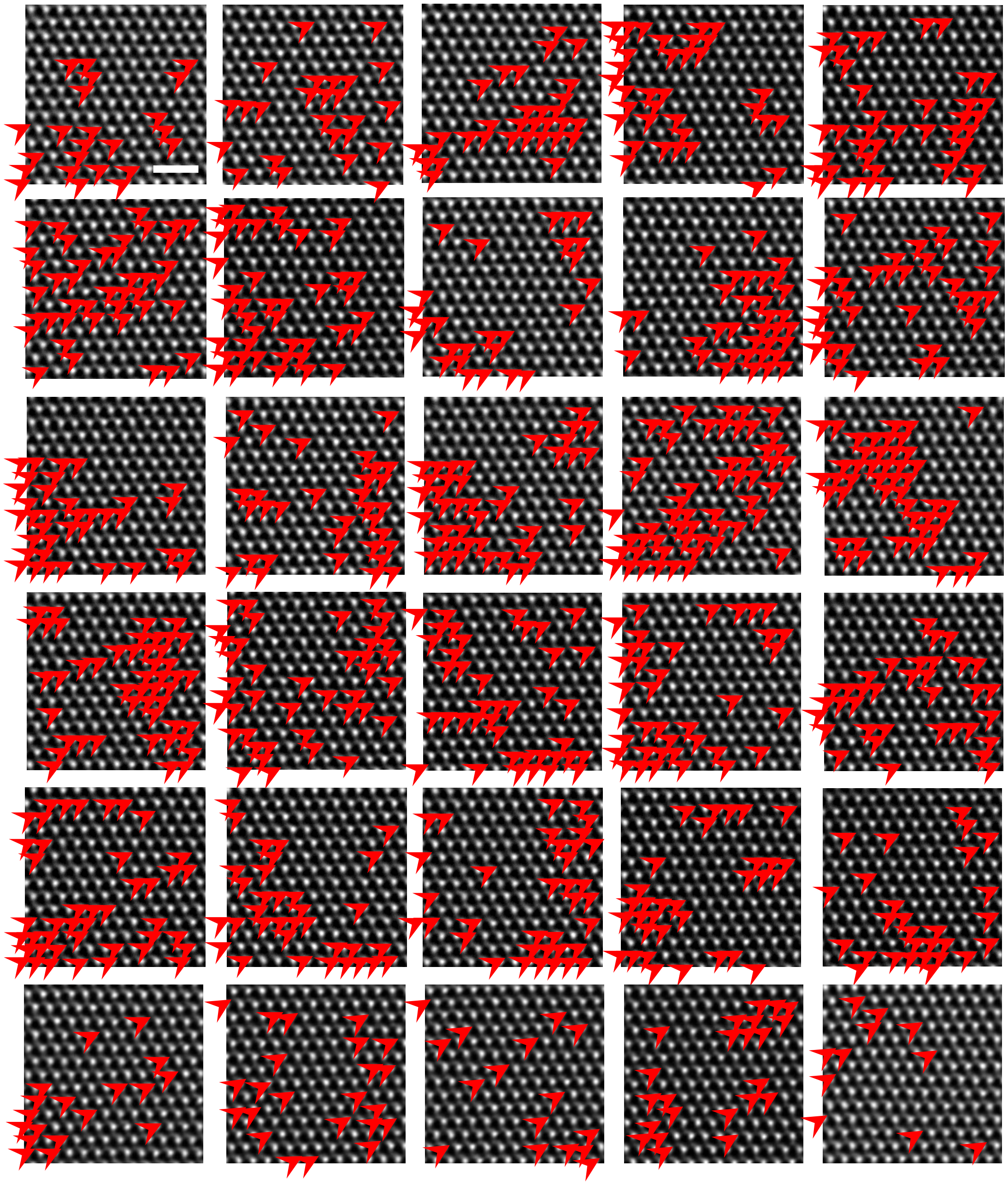

Supplementary Fig. 13 | Statistics of sulfur vacancy distribution in monolayer WS_2 after pretreatment in H_2O_2 solution for 25 min. Scale bar: 1 nm.

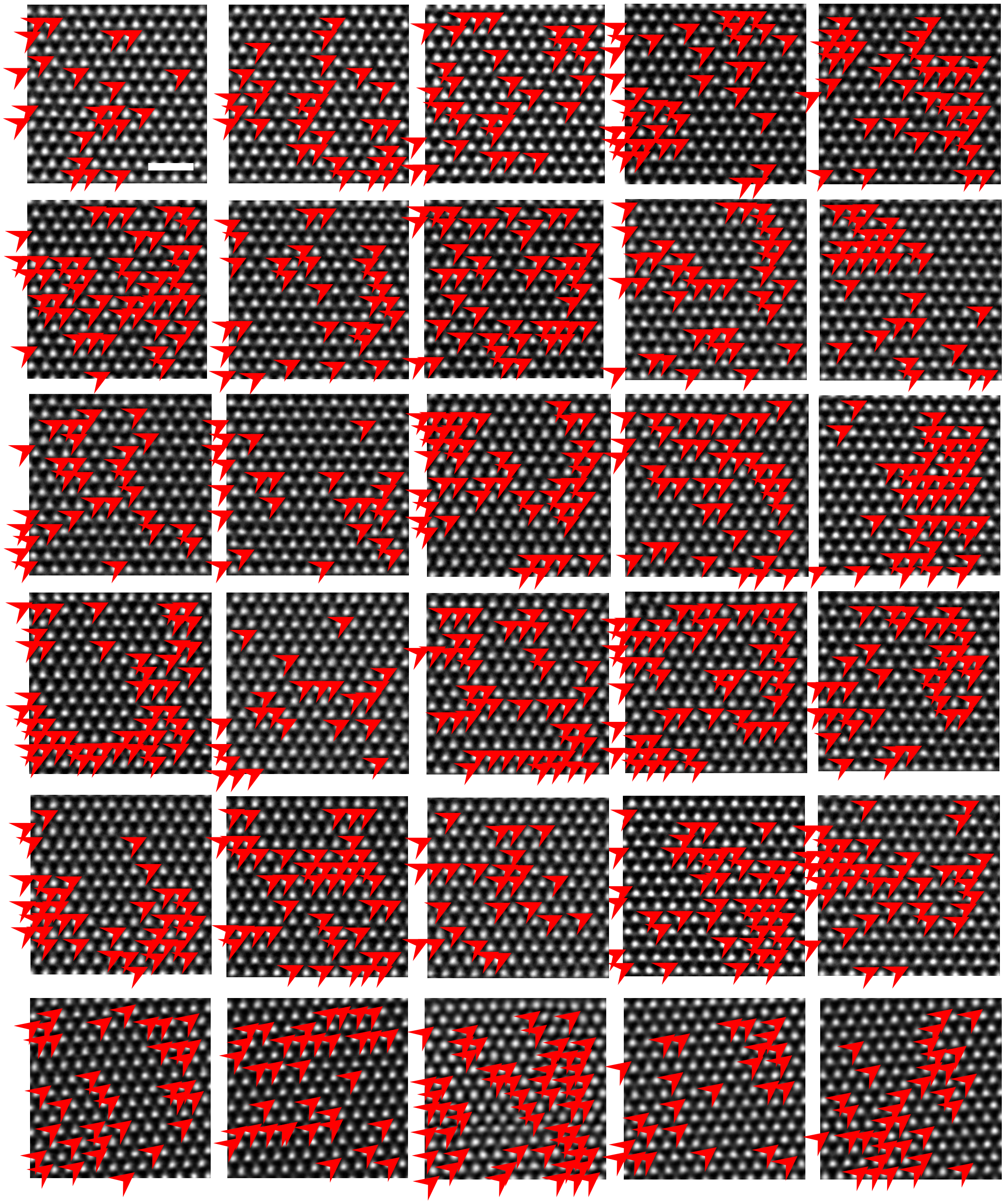

Supplementary Fig. 14 | Statistics of sulfur vacancy distribution in monolayer WS_2 after pretreatment in H_2O_2 solution for 30 min. Scale bar: 1 nm.

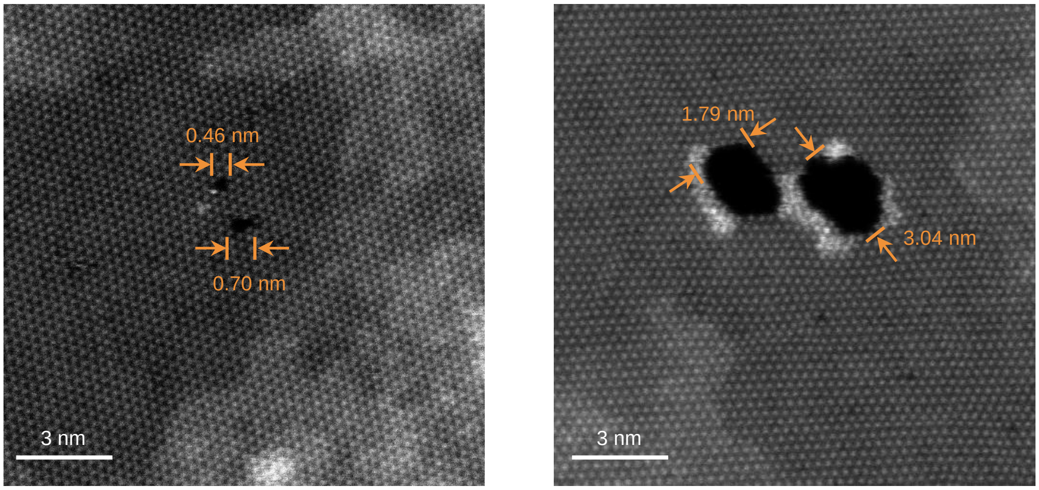

Supplementary Fig. 15 | STEM images of WS_2 (t_{pt}=30 min) containing small openings.

9. Raman characterization for excluding structural phase change

It is well known that structural phase change in TMDC materials is prone to take place under uniformly intercalative (e.g., Li and K atoms) or substitutional (e.g., Re, Tc and Mn atoms) doping that leads to *collective atomic displacements* (i.e., highly synchronous motion of atoms). In our case of vacancy generation by H_2O_2 treatment, the sulfur vacancies are randomly distributed and are relatively *separated from each other*. Also, the doping level is relatively low (see the electronic characterization below). Hence, there is little chance to initiate a highly synchronous displacement of collective atoms to trigger a well-defined phase change.

To exclude the possibilities of structural phase change and flake-to-flake dependence, we performed corresponding Raman characterization on WS_2 samples treated under various conditions. Supplementary Fig. 16a shows the typical Raman spectrum for a 30-min treated WS_2 sample, in which no any sign of structural phase change is observed. The two peaks located at 357 and 418 cm^{-2} can be assigned to the E_{2g}^{1} and A_{1g} modes from the 2H phase, while no remarkable modes from 1T or 1T' phase (indicated by the red and blue arrows) are detected. We also checked the point-to-point variation by performing Raman mapping over broad areas and found no evidence of phase change, as given in Supplementary Fig. 16b.

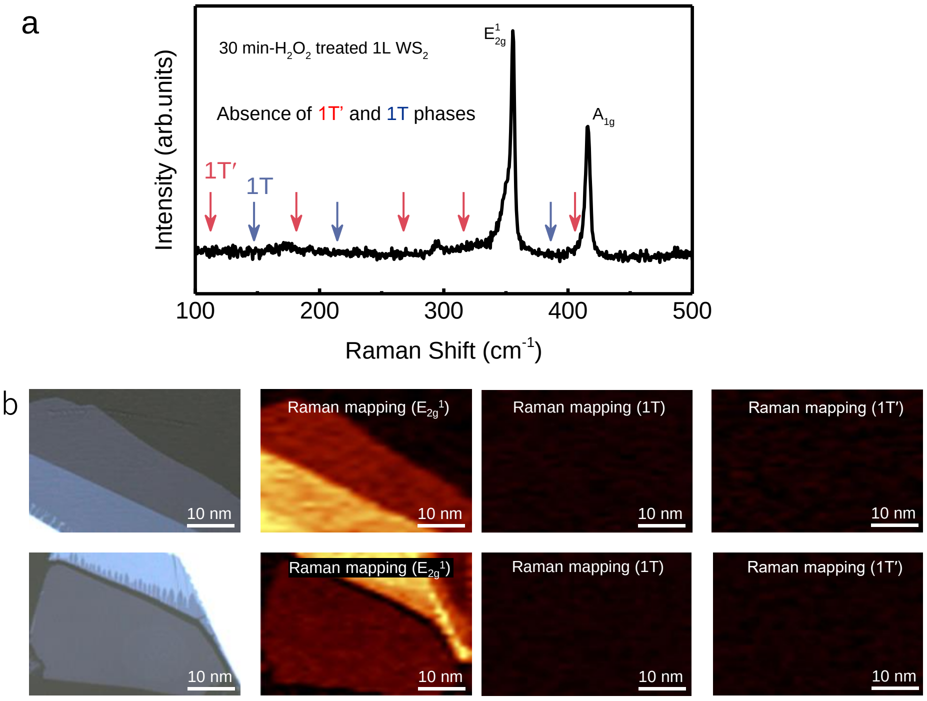

Supplementary Fig. 16 | Raman spectrum and spatial mapping to rule out the presence of possible structural phase change to 1T or 1T' phase.

In Supplementary Fig. 17, we further checked the flake-to-flake variation by comparing the Raman spectra between multiple WS_2 samples treated with H_2O_2 for different durations. A consistent observation was made. In combination of the electronic and spectral characterization, it is therefore safe to exclude the presence of structural phase change due to electronic doping or collective atomic displacement.

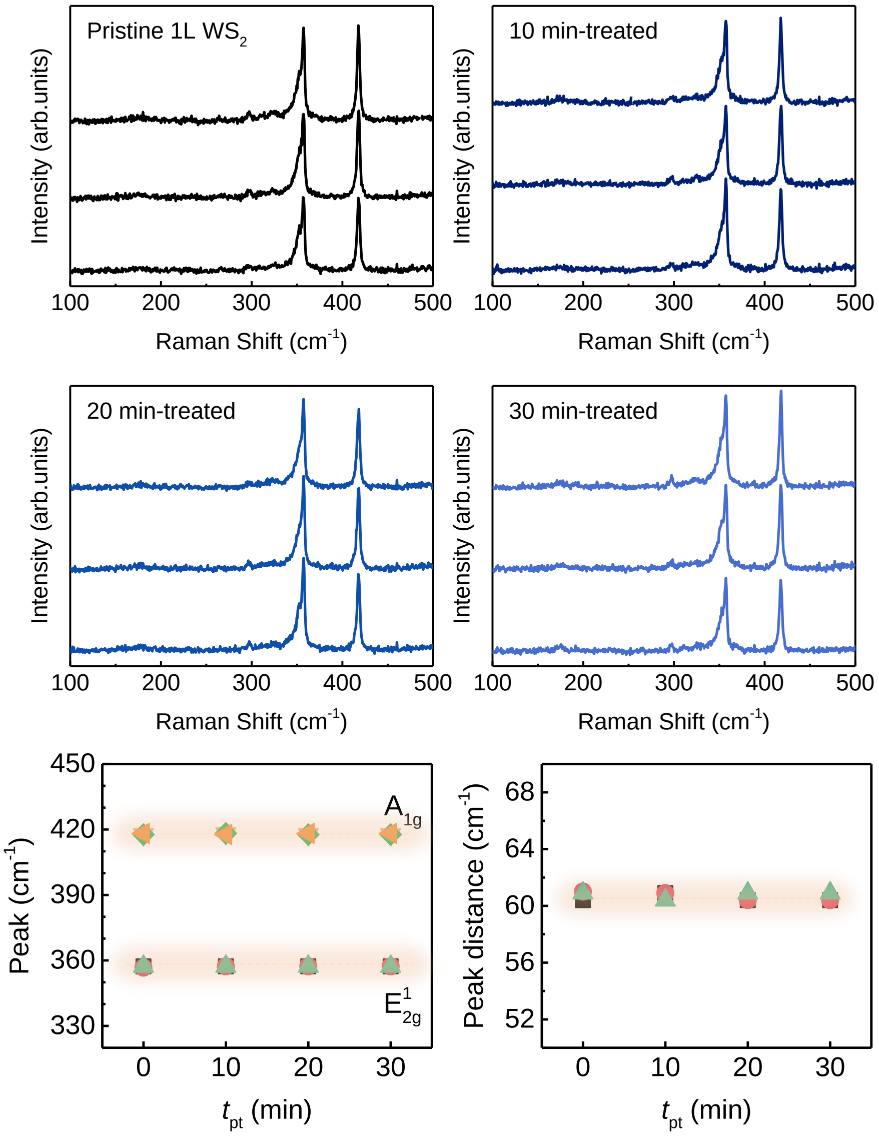

Supplementary Fig. 17 | Absence of possible Raman shifts in WS_2 flakes treated in H_2O_2 treatment for different durations due to vacancy generation.

Convincing evidence against phase change can be obtained from the preservation of the semiconductive characteristic, because the TMDCs of a 1T or 1T' phase is generally metallic. Supplementary Fig. 18a shows the transfer curves for a same WS_2 transistor successively treated for different durations from 0 to 30 min. The device exhibits a semiconductive characteristic under all conditions, although the gradual degradation of performance due to the increase of vacancies. Furthermore, the realistic doping levels after H_2O_2 treatment were extracted (under the criterion of 1p A as the off state) and shown in Supplementary Fig. 18b, which indicates only a moderate n-doping effect (~4×10^{12} cm^{-2}), in contrast to the presence of a high density of sulfur vacancies (~3×10^{14} cm^{-2}). We deduce that the sulfur vacancies are likely passivated by oxygen or other light chemical groups that are invisible in STEM. With this, we further guess that the residual brightness intensity in the sites of V_{2S} (Supplementary Figs. 7e and 32e) stems from those light chemical groups that are grafted to the atoms adjacent to the vacancies.

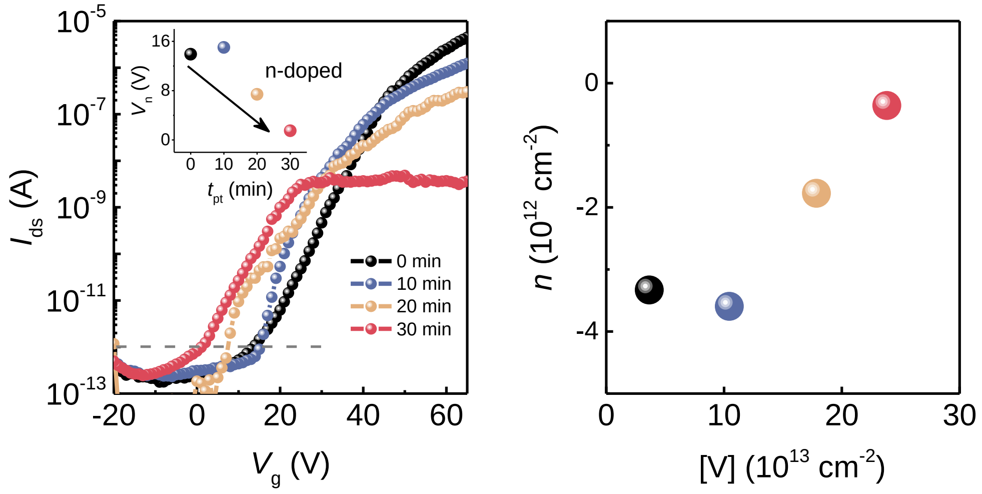

Supplementary Fig. 18 | Transfer curves for a WS_2 FET channel treated with H_2O_2 solution for different durations and corresponding doping effect.

10. Vacancy introduction revealed by XPS spectra

We performed XPS characterization to quantitatively estimate the densities of sulfur atoms removed after defect engineering in H_2O_2 soaking. In total, four WS_2 monolayers under different t_{pt}s from 0 to 30 min were adopted. The untreated sample (t_{pt} = 0 s) was used as reference and extended pretreatment durations of 10–30 min were employed for other samples to check the effect of after defect engineering.

Supplementary Fig. 19a–d shows the XPS spectra for the W and S elements and corresponding Gaussian fittings, to estimate the atomic ratios between S and W. The main doublets from W are excited from its 2*f* orbit and located around 33.5 and 35.7 eV, while the doublets of S are from 2*p* orbit and located around 163.0 and 164.2 eV. Although the absolute magnitudes of detector counts vary with sample sizes, the relative intensity ratios between elements within each sample can be used to analyze the densities of removed sulfur atoms after defect engineering. In Supplementary Fig. 19e, the extracted S/W atomic ratio is plotted versus t_{pt}. It linearly decreases from 1.97 to 1.56 as t_{pt} increases from 0 to 30 min, indicating that the introduction of S vacancies is roughly proportional to t_{pt}. Accordingly, the percentage and density of S were also estimated and given in Supplementary Fig. 19f,g.

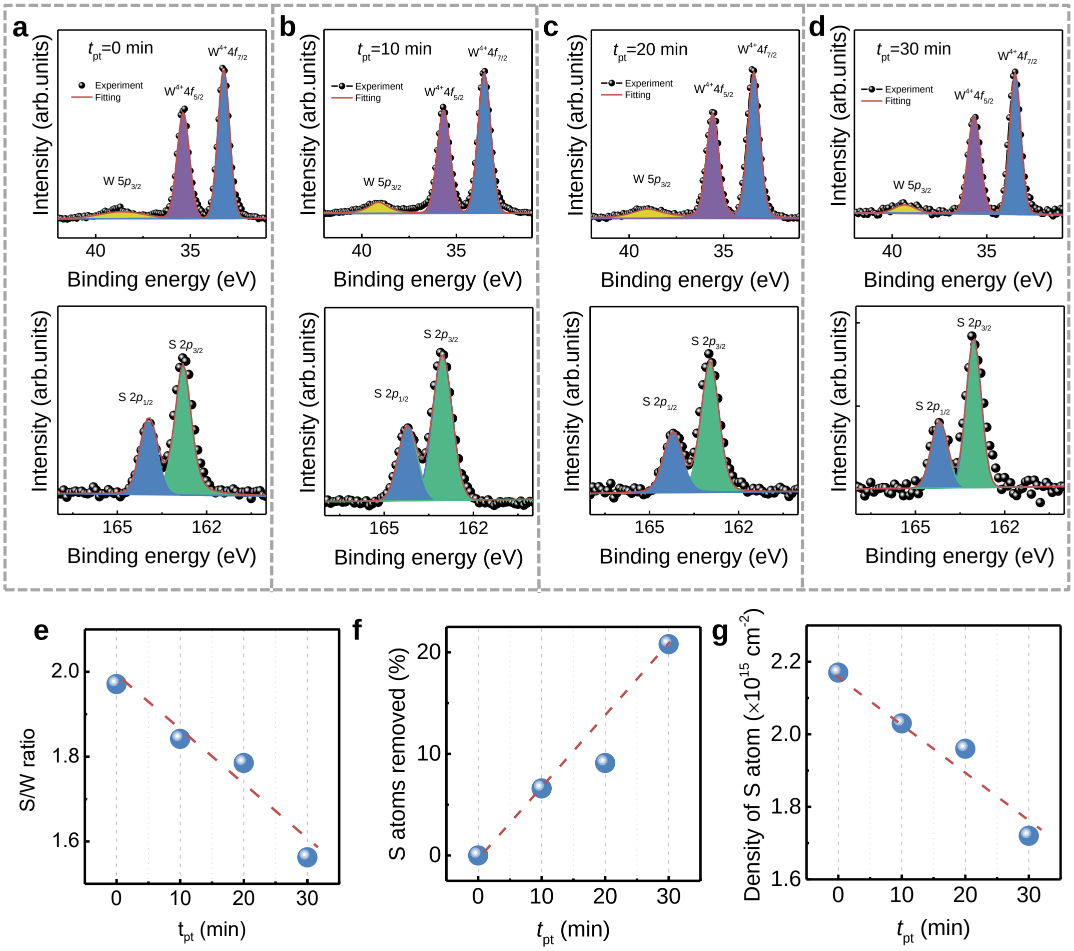

Supplementary Fig. 19 | Engineering of sulfur vacancies in H_2O_2 pretreatment as revealed from XPS spectra. a-d, XPS spectra for 1L WS_2 at different pretreatment duration (t_{pt}) in H_2O_2 solutions for 0 to 30 min. The quantities of W and S elements are analyzed from the weights of the related peaks via Gaussian fittings. **e**, Extracted S/W atomic ratio versus t_{pt}., which decreases with increasing t_{pt}, indicating the loss of S atoms after pretreatment. **f**, Percentage of S atoms removed relative to the pristine lattices. **g**, Density of preserved S atoms versus t_{pt}.

11. Overview of extracting oxidation length for statistics

Supplementary Figs. 20 and 21 show two representative examples for extracting oxidation length in partially oxidized WS_2 and MoS_2 for statistics. The lengths of oxidation are extracted by drawing multiple progression lines, at reasonably large intervals, along the normal direction along the original flake edges and relevant reaction boundaries. For each oxidation condition, we collected relevant lengths of oxidation data from about 30–120 locations from 3–5 samples.

As can be seen in Supplementary Fig. 20a, the frontal reaction boundaries are not necessarily shape in edge, that is, they are not parallel to the original flake edges, which is a natural consequence of fluctuation in oxidation rates due to non-uniform distribution of lattice defects. In most cases, the histogram from one flake (e.g., Supplementary Fig. 20a) cannot be well fitted with the Poisson statistical function. However, the histogram would be in a good statistical shape when the results from more flakes (Supplementary Fig. 20b,c,d) are merged (Supplementary Fig. 20e). Thus, the number of samples would be slightly increased if large dispersion is encountered at different locations or samples, to reduce the overall uncertainties.

In the experiment, more than 400 samples were tested in total. On average, it took one day for characterizing one sample, including mechanical exfoliation, pretreatment, collection and processing of photooxidation data. Roughly, one and a half year was taken only to collect the photooxidation data, not to mention the time for other data and analyses, such as time-consuming crystallographic characterization on [V] by STEM.

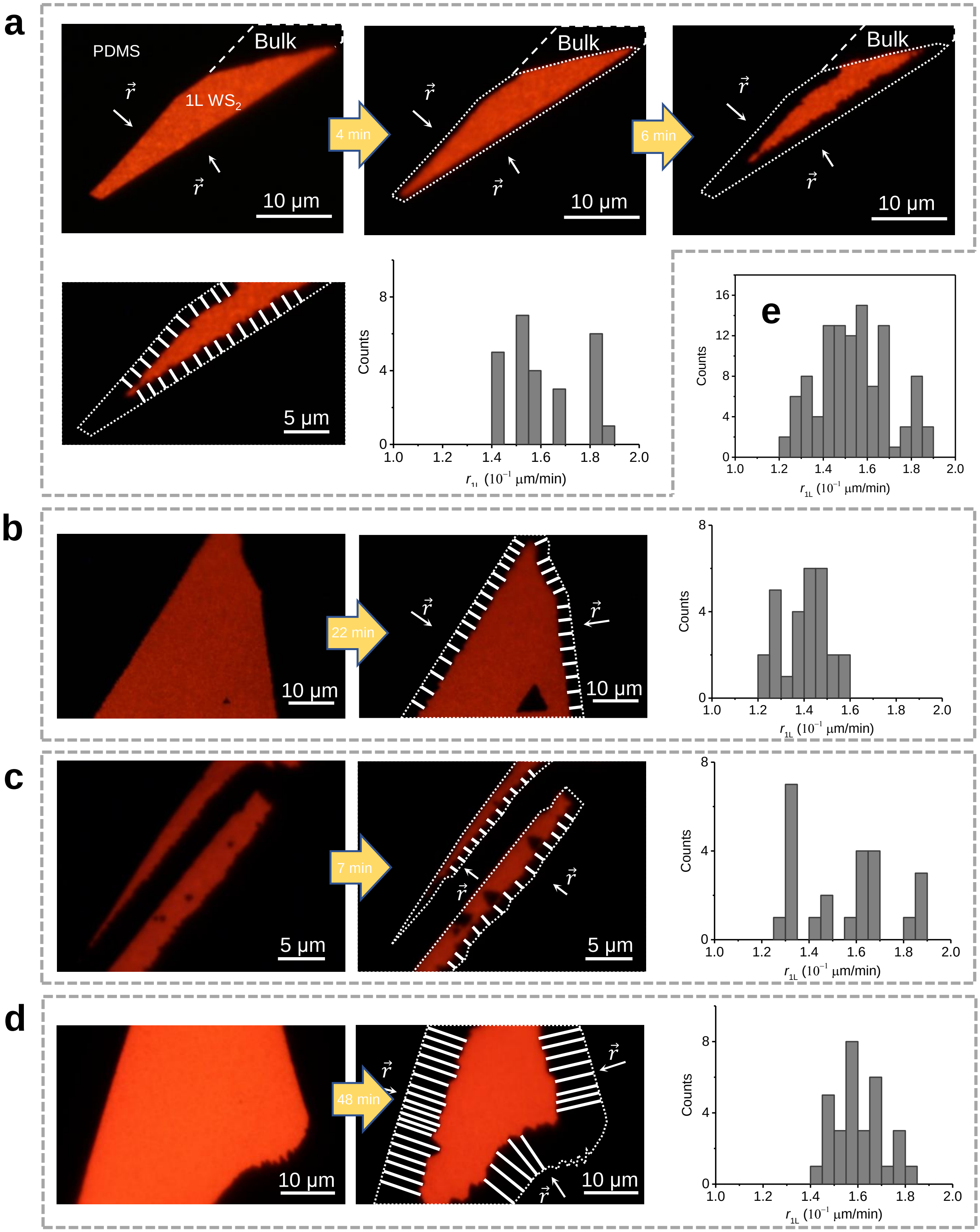

Supplementary Fig. 20 | An overview of statistical process for lateral oxidation rate in multiple WS_2 samples that are photo-oxidized under a same condition. The crystallographic and environmental conditions are [V] = 2.75×10^{14} cm^{-2}, T = 26 ˚C, and RH = 60%. **a**, Serial PL images at different oxidation stages with showing the reaction lengths (solid white bars) at different peripheral locations. **b-d**, Repeated procedures for another three samples. **e**, Histogram with merged data that exhibits a shape of Poisson distribution.

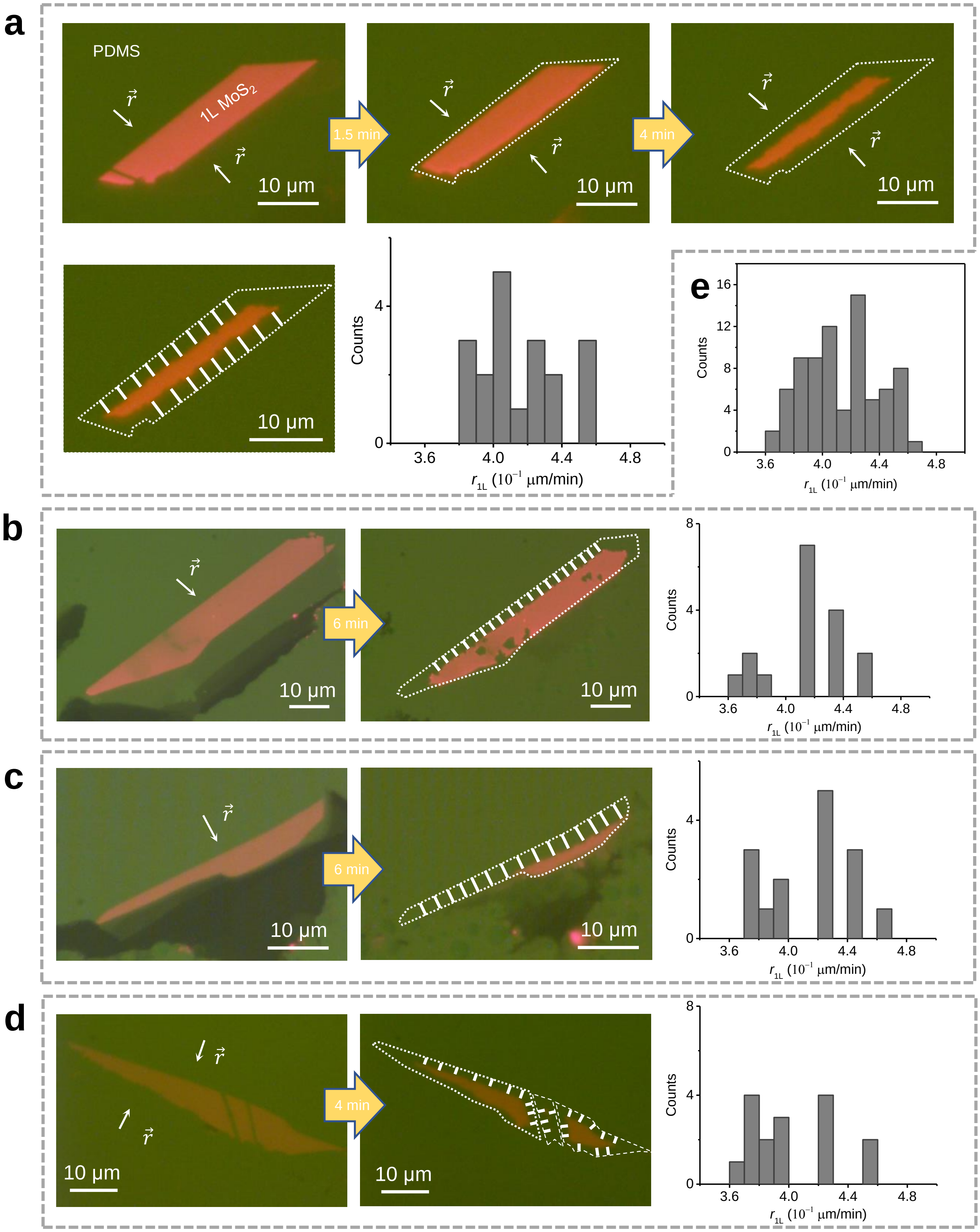

Supplementary Fig. 21 | An overview of statistical process for lateral oxidation rate in multiple MoS_2 samples that are photo-oxidized under a same condition. The crystallographic and environmental conditions are [V] = 7.1×10^{13}cm^{-2}, T = 20 ˚C, and RH = 60%. **a**, Serial PL images at different oxidation stages with showing the reaction lengths (solid white bars) at different peripheral locations. **b-d**, Repeated procedures for another three samples. **e**, Histogram with merged data that exhibit an average value of 0.42 ± 0.04 μm/min.

12. Simulation on photothermal effect

To check the photothermal effect and investigate the local temperature of the samples under the illumination of a power density of 6 W/cm^2, a commercial software was employed to simulate the T rise during illumination. The net optical absorptivity values for monolayers and underlying PDMS substrates are set to 5% and 0, respectively. The thermal conductivities are adopted as 116.8 and 0.16 W/(K·m) for WS_2 and PDMS substrate[3], respectively. The environmental T base is set at 300 K. As shown in Supplementary Fig. 22, the steady-state T value is only 300.08 K under the 6 W/cm^2 illumination. Only a small T rise of 0.08 K is observed.

To further verify the reliability of the software, we also simulated the case 1L WS_2 on SiO_2/Si substrates (9×10^6 W/cm^2) reported in reference[4]. The simulation shows a consistent local T of 1998 K with the reference, indicating the reliability of the software.

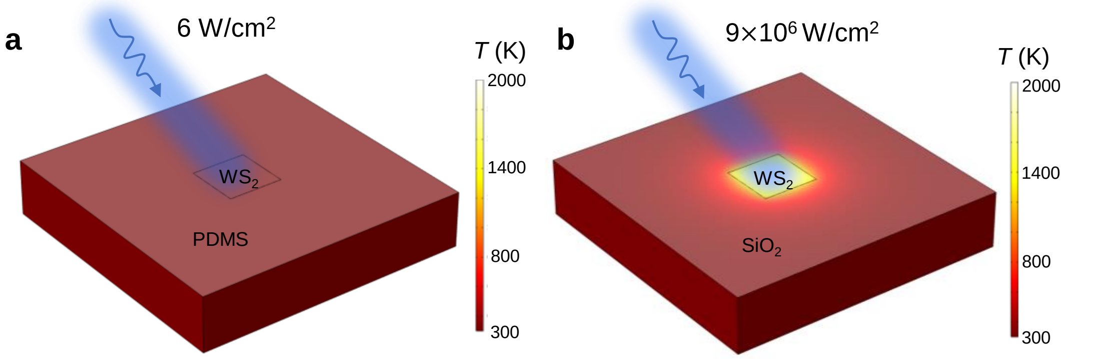

Supplementary Fig. 22 | Spatial distribution of temperature. Simulation of the 2D temperature field under two steady states of different power densities (**a**: 6 W/cm^2; **b**: 9×10^6 W/cm^2). In the 300 K environment, the local temperature is only 300.08 K for 1L WS_2 on PDMS substrate under 6 W/cm^2 illumination, but the local temperature rises to 1998 K under 9×10^6 W/cm^2 illumination (SiO_2 as substrate).

13. Service reliability due to degradation

As an application of the knowledge of oxidation kinetics, the time-dependent degradation behavior of electrical performance in 1L WS_2 was recorded under different humidity conditions. Three RH levels, including < 0.1 ppm (in glovebox), 30% and 70%, were employed in experiment to check the realistic effect of humidity, as well as the existence of ~46% critical threshold for distinguishing the dry and wet oxidation mechanisms.

Supplementary Fig. 23a–c, shows the degradation of transfer curves along time for three FETs consisting of 1L WS_2 channels, where the maximum durations are 6480, 144 and 168 h, respectively. In the case of the oxygen-and water-free surrounding in glovebox, the oxidation rate is rather low and only ~20% reduction in device current is recorded in the 9 months (6480 h) long storage duration (Supplementary Fig. 23d). Supplementary Fig. 23e summarizes the current degradation for all the three FETs, which reveals a general trend of exponential decay in current with storage time. Clearly, the degradation rate under different RH surroundings follows the sequence: 70% > 30% > glovebox (<0.1 ppm). After ~150 h storage, the residual current ratios are about 5%, 50%, 99%, respectively. The slow degradation ratio in glovebox confirms the crucial roles of oxygen and humidity in oxidation, while the 10-fold difference in residual current between the 30% and 70% RH surroundings corroborates the existence of distinct dry and wet oxidation mechanisms that are separated by the ~46% RH threshold.

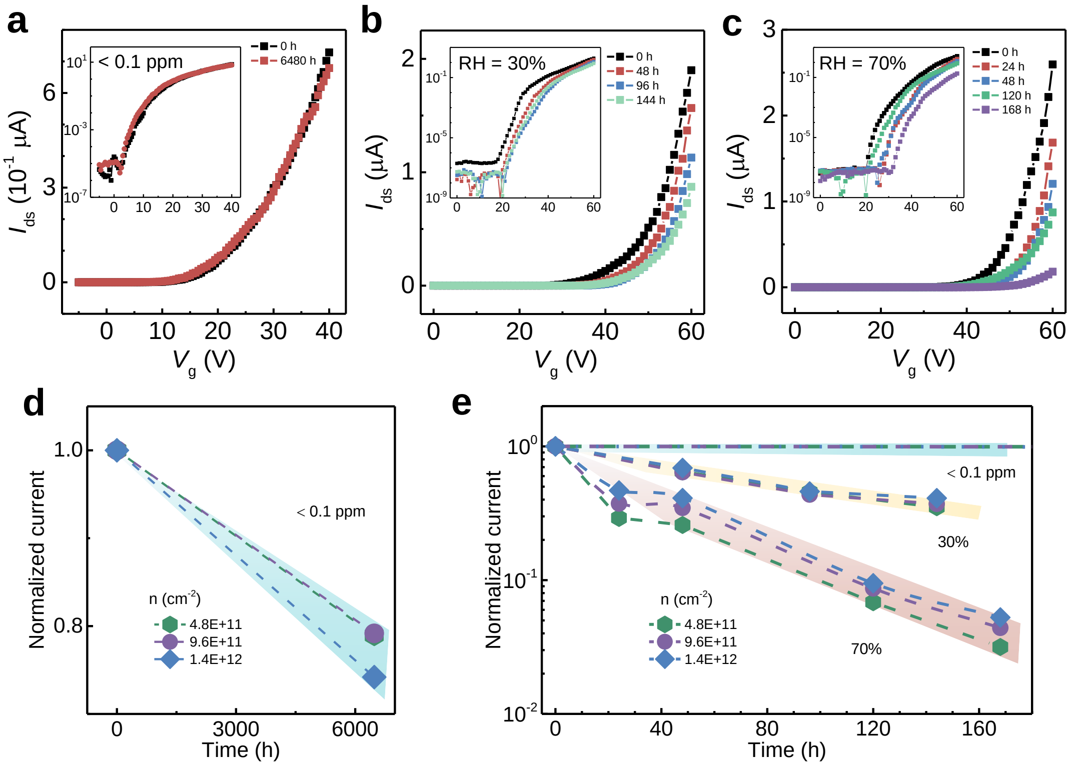

Supplementary Fig. 23 | Service expectancies of 1L WS_2 under different humidity levels evaluated by electronic performance. a-c, Evolution of transfer characteristics with time. under relative humidity levels at (**a**) less than 0.1 ppm (in glove box), (**b**) 30 % and (**c**) 70 %. **d** and **e,** Normalized current versus time at varied humidity levels. A tendency of exponential decay is observed in all cases, which provide a way for prediction of service life.

14. Detecting activation threshold in photon energy

To further prove the activation threshold in photon energy, we designed an experiment by using different laser sources at different stages, with the designed sequence: 450, 650, and 638 nm. The inactive 650-nm laser was intentionally arranged in the middle to ensure the convincingness of this experiment. As shown in Supplementary Fig. 24, oxidation behavior is excited in WS_2 at stages I (450nm, 2.76 eV) and III (638 nm, 1.94 eV), it is inactive, in the middle, at stage II (650 nm, 1.91 eV). The result implies the threshold energy ~1.91 eV.

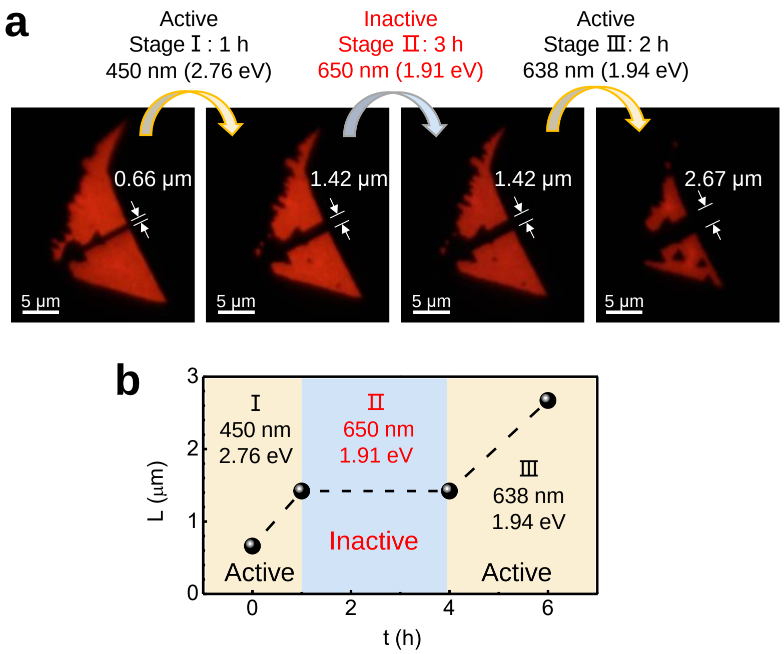

Supplementary Fig. 24 | Photo-oxidation of 1L WS_2 under different photon energies. a, PL images for 1L WS_2 under designed sequential illuminations: I) 450 nm for 1 hour, II) 650 nm for 3 hours, III) 638 nm for 2 hour. **b**, Extracted oxidation length (*L*) versus illumination times (*t*). WS_2 is inactive only in the middle stage. Thus, 650 nm is energetically insufficient to trigger fast photo-oxidation.

15. Control experiment in glovebox

Oxygen and moisture molecules are reported the most key species engaged in the oxidation process.[5] We also performed a control experiment by illuminating pretreated WS_2 sheets in an oxygen- and moisture-free glovebox (both levels < 0.1 ppm) to check their roles.

In order to strictly control the concentrations of water and oxygen absorbates from surroundings, the WS_2 sheets were exfoliated and pretreated (H_2O_2 solution, 30 min) *in*

situ in separated glovebox chambers. Meanwhile, the PDMS substrates used were heated in advance to desorb molecules on its surface. Supplementary Fig. 25 shows the optical images for a 1L WS_2 before and after 24-hour illumination in the glovebox. No noticeable morphologic change is seen from the reflection images. This result indicates that a sole light illumination cannot trigger the oxidation reaction and confirms the crucial roles of oxygen and moisture in the oxidation process.

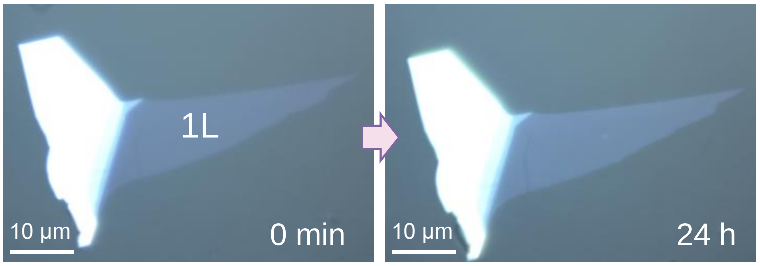

Supplementary Fig. 25 | Negligible oxidization in a water- and oxygen-free glove box after 24-h irradiation, indicating the crucial roles of ambient water and oxygen in the oxidation process. The 1L WS_2 sample was pretreated in H_2O_2 solution for 30 min before illumination and the levels of water and oxygen were less than 0.1 ppm.

16. Reaction rate versus substrate hydrophilicity

Previous studies have demonstrated that the surface condition of substrates has a direct impact on the chemical reactivity of the materials supported. Here we checked the effect of hydrophilicity of PDMS substrates on r_{1L}. The PDMS is intrinsically a hydrophobic polymer. We intentionally tune its hydrophilicity by varying the exposure time in different high-energy plasma surroundings and the overall surface hydrophilicity was evaluated with the contact angle of water drops on them. The contact angle can be tuned from primitively hydrophobic ~110° to hydrophilic ~15° after a high dose exposure of Ar plasma. The surface modification changes the surface condition and moisture density absorbed, which hence remarkably tune the reaction rate. As shown in Supplementary Fig. 26, r_{1L} increases monotonously with the contact angle exhibited by the surface-modified PDMS substrates. This trend can be attributed to the additional reaction sites at the bottom surfaces that facilitate charge transfer during reaction, in line with the crucial role of moisture on oxidation.

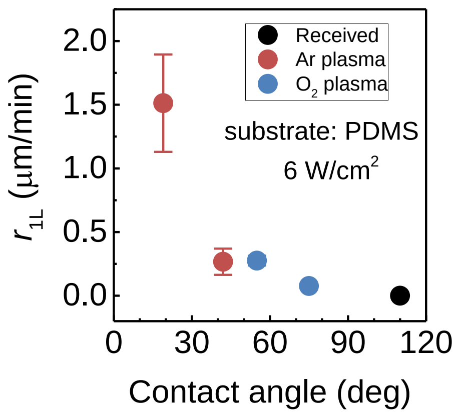

Supplementary Fig. 26 | Correlation between reaction rate and contact angle of substrates. The O_2 or Ar gas plasma was used to change the hydrophilic property (i.e., contact angle) of the PDMS substrates. An inverse correlation between r_{1L} and contact angle was observed. Such a tendency can be attributed to the additional reaction sites at the bottom surfaces for charge transfer during reaction, which corroborates the crucial role of surrounding moisture on oxidation. Standard deviations are used as the error bars.

17. Selection of humidity forms

When formulating r_{1L}, we carefully selected the form of the parameter humidity (absolute or relative values, AH or RH) by comparing the plots of r_{1L} versus AH and RH. Although adopting AH as the parameter to formulate r_{1L} is physically more meaningful than RH, we found that adopting RH would result in a concise algebraic form accounting for all the T values from 20 to 26˚C. When adopting RH as the humidity parameter, all the curves converge at a critical threshold point around RH ~ 46% (Figs. 2n and 4d). Hence, the parameter RH is naturally adopted when rationalizing the quantitative relationship between r_{1L} and humidity.

18. UPS characterization

To provide direct evidence, the ultraviolet photoelectron spectroscopy (UPS) technique was used to determine the realistic energy levels for 1L WS_2 and MoS_2, as shown in Supplementary Figs. 27 and 28. In Supplementary Fig. 27, we first verify the reliability of UPS for the Au calibrator and monolayer semiconductors. To this end, we prepared as-grown and p-doped 1L CVD WS_2 and MoS_2. A 40 nm gold (Au) film is electrically connected to all the disulfide samples and steel stage (Supplementary Fig. 27a) to level the chemical potentials (Fermi levels) of all. The signal form the Au film is also used as the calibration. The UPS was operated under the static charge compensation mode to ensure

the detection of the weak signals from WS_2 and MoS_2 monolayers; the chamber vacuum was kept at 10^{-4} Pa for at least 48 hours for surface desorption.

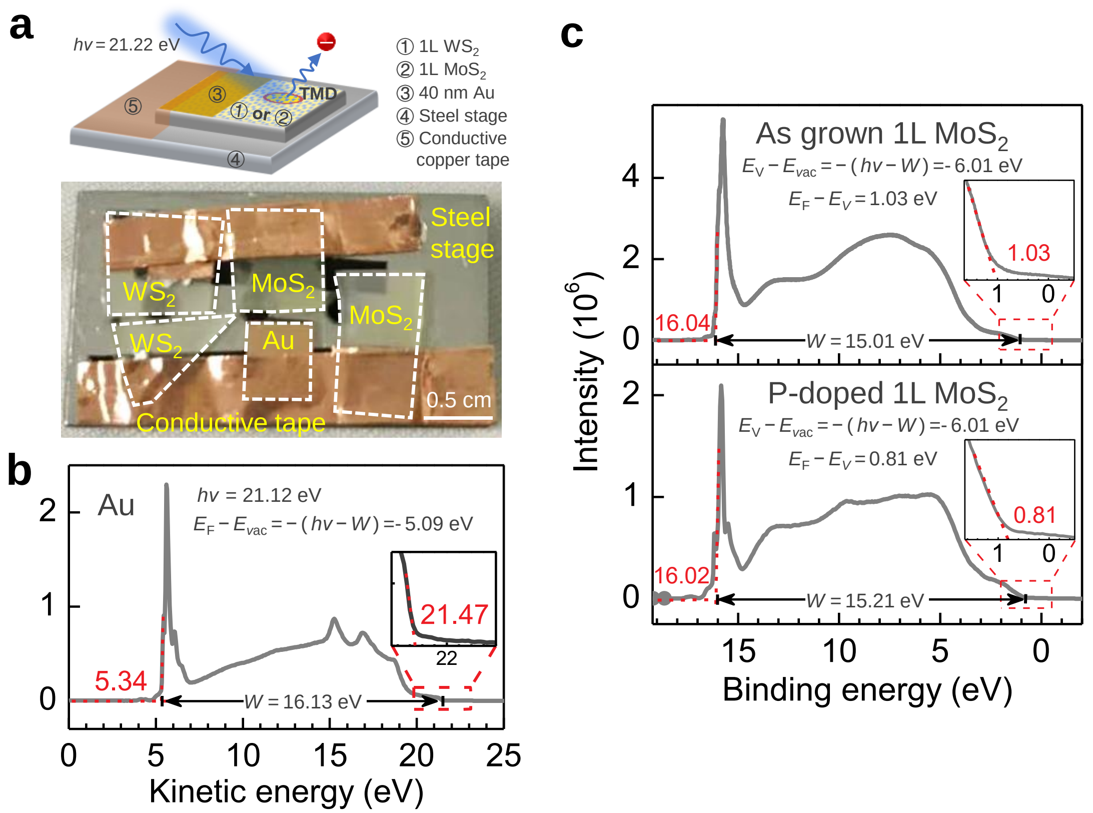

Supplementary Fig. 27 | Check on accuracy and sensitivity of UPS analysis in determining the energy levels of a metal and a monolayer semiconductor. a, Diagram and real optical image for samples mounted on the steel stage for UPS measurement. **b,** UPS spectrum of Au calibrator. Inset: Enlarged regime around the Fermi edge. The work function of Au can be extracted from the difference between the excitation energy (hv = 21.12 eV) and the spectral width, W (calculated from cut-off region to Fermi edge). **c**, Comparison of the UPS spectra for as grown and p-doped 1L MoS_2. A small E_F shift around 0.18 eV is observed after doping. All spectra were collected under the electrostatic charge compensation mode to maximize the signal intensity.

Supplementary Fig. 27b shows the raw kinetic spectrum for the Au calibrator. The cutoff and Fermi edges are located at 5.34 and 21.47 eV, respectively. These features give rise to a spectral width of 16.13 eV and an E_F of 5.09 eV, agreeing well with the recognized work function of 5.1 eV for Au (111).

We also verify the applicability of UPS on the as-grown and p-doped 1L CVD MoS_2. The p-doping was fulfilled by evaporating a trace of an electron-drawing small molecule bis(trifluoromethylsulphonyl)imide, TSFI, on the MoS_2 surfaces, to mimic the effect of

absorbed aqueous oxygen (which is unstable in the UPS vacuum chamber). Supplementary Fig. 27c compares the values of energy levels for the 1L CVD MoS_2 before and after p-doping. For the as-grown sample, the conduction band (E_V) is located 6.01±0.04 eV below the vacuum energy (E_{vac}), which is located just slightly higher than the HSE06 calculated value[6] (6.27 eV), indicating the reliability of the HSE06 method. From the Fermi edge, we deduce that E_F-E_V = 1.03±0.04 eV, indicating the neutral or slightly n-doping nature of the as-grown samples after considering the ~2 eV bandgap. In contrast, the Fermi level goes to the level at 0.81±0.04 eV above E_V, featuring ca. 0.2 eV lowering after p-doping. The sensitivity in detecting the shift of E_F by p-doping further confirms the high reliability of UPS for characterizing the energy levels of monolayer semiconductors.

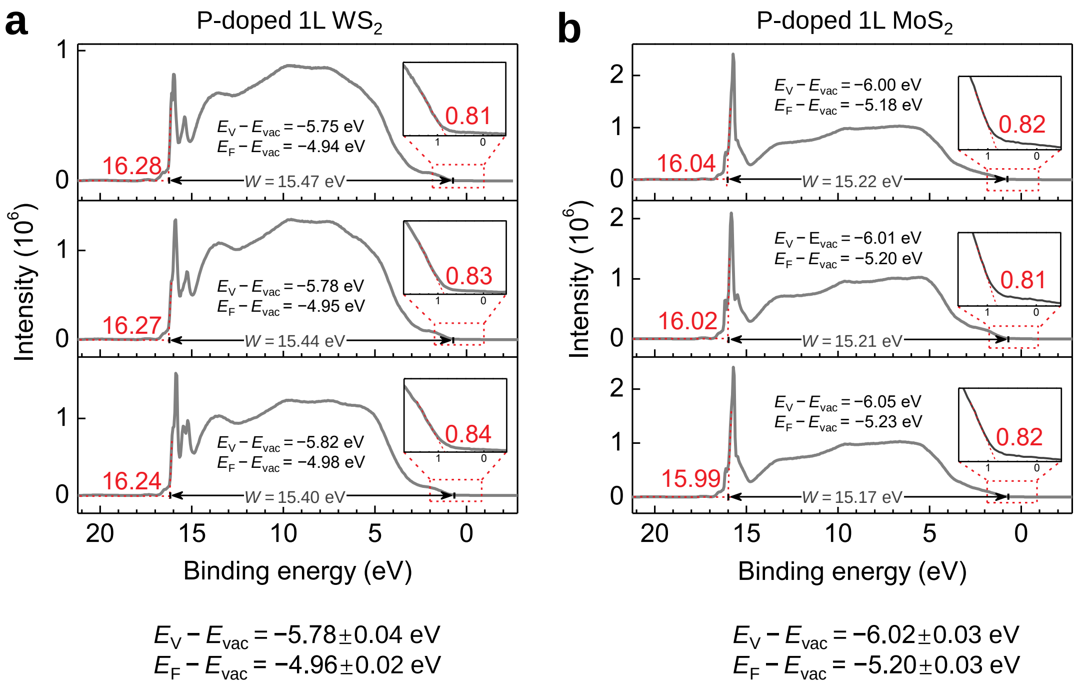

Supplementary Fig. 28 | Check on reproducibility of the UPS facility from multiple locations in 1L WS_2 and MoS_2. a, Spectra collected from 3 locations in WS_2. **a,** Spectra collected from 3 locations in MoS_2. The deviation is less than 0.04 eV for every energy scales.

Furthermore, we checked the sample uniformity by collecting the UPS spectra from three different areas in the doped 1L WS_2 and MoS_2. As shown in Supplementary Fig. 28, the spectral shape and characteristic energy positions are consistent with each other for each sample. The energy levels estimated for 1L WS_2 and MoS_2 are summarized in Supplementary Table 1 and are also given in Figs. 3e and 4g. There is ~0.2 eV difference

in E_F positions between the two. The UPS data support the band bending picture that accounts for the difference in the additional energy barrier between WS_2 and MoS_2 during charge transfer.

Supplementary Table 1 Summary of energy levels for p-doped 1L WS_2 and MoS_2.

	E_v - E_{vac}	E_F - E_v	E_F - E_{vac}
p-doped 1L WS_2	-5.78±0.04	0.83±0.02 eV	-4.96±0.02 eV
p-doped 1L MoS_2	-6.02±0.03	0.81±0.02 eV	-5.20±0.03 eV

19. Reaction paths and band diagram

By comparing the magnitudes of simulated E_a values with experiment, we can first rule out two reaction paths pertinent to defect-free lattices. As shown in Fig. 3b, the barriers for the dissociation of oxygen species amount to 2.89 and 1.66 eV in the dry and wet conditions, respectively, where the oxidants originate from the molecular (O_2) and anionic (O_2^-) oxygen. The simulated energies are larger than the experimental values, being 2.5 eV and 1.4 eV for the dry and wet conditions, respectively (inset of Fig. 2p).

We then shift to two other reaction paths related to defective lattices where sulfur vacancies dominate (Fig. 3c). In the dry condition, molecular oxygen serves as the main oxidant and there are two barriers of 0.87 and 2.3 eV in the energy landscape. In contrast, the reaction barrier is reduced to 0.92 eV in the wet condition, where the highly active O_2^- ions act as oxidants. The lowered activation energies are more reasonable to explain the experimentally extracted E_a values. Evidently, the magnitude of E_a is primarily determined by the exact type of oxidation agents generated under different humidity conditions.

Supplementary Fig. 29 shows the formation of realistic band diagram in the electrochemical reaction between 1L WS_2 and absorbed aqueous oxygen. Before humidity condensation onto WS_2 (i.e., under dry surroundings), all the relevant energy levels are flat without any bending, as shown in Supplementary Fig. 29a. According to the UPS results, the positions of E_v ~ 5.8 eV and E_F ~ 4.95 eV below the vacuum energy level (E_{vac}) after considering the ~ 2 eV energy gap. Since the redox energy of ($E_{F,redox}$) is 5.3 eV below E_{vac}, a potential difference around 0.35 eV occurs between WS_2 and aqueous oxygen, which leads to a upward band bending of 0.35 eV and an extra excitation barrier for charge transfer at the interface.

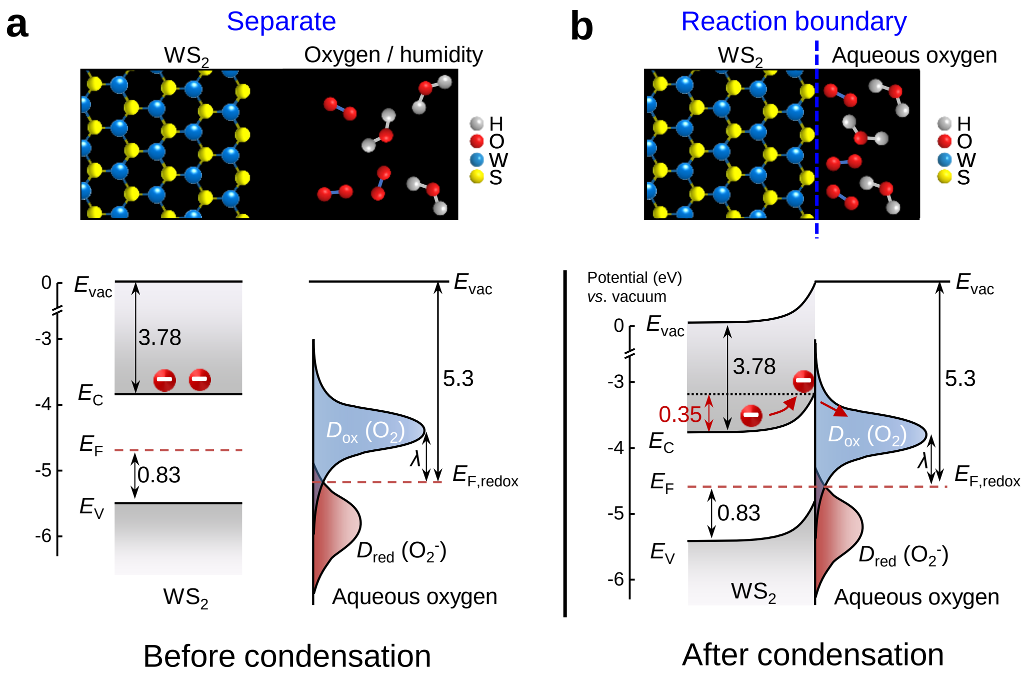

Supplementary Fig. 29 | Formation of band diagram in electrochemical reaction between WS_2 and aqueous oxygen. a, Individual band structures before condensation of aqueous oxygen on 1L WS_2. **b**, Realistic band diagram formed in electrochemical oxidation accounting for the band bending, where an extra barrier of 0.35 eV is formed.

20. Defect engineering via an alternative oxidant

Besides the H_2O_2 solution, we also tested the feasibility of using other oxidants for defect engineering. We tried a Lewis acid, bis(trifluoromethane)sulfonimide (TFSI), as the oxidant for quick pretreatment. This material is known to be a strong p-dopant on 2D materials,[7] and is also proven a medium for PL enhancement.=[8,8] In this trial, the 1L WS_2 was mechanically exfoliated on sapphire substrates. The TFSI powders were dissolved in nitromethane to make a 0.2 mg/ml solution. The TFSI solution was then drop-cast onto the sample and kept for 5 min at room temperature.

Supplementary Fig. 30a shows the PL traced oxidation process. Similar to the samples pretreated by H_2O_2, this sample also exhibits peripheral oxidation behavior with the reaction fronts propagating from the edges to the central area. The PL signals are remarkably enhanced around the reaction fronts, implying a strong p-doping effect and modulation on concentration of neutral excitons in WS_2 by TFSI. We intentionally terminated the oxidation and trace midway at 33 min, so that a part of unoxidized area was preserved. Then, we employed AFM to check the whole sample region, including both the oxidized and unoxidized areas. Supplementary Fig. 30b shows the AFM image covering

the two areas. In the reflection images, the oxidized areas are optically invisible because of their insulating nature, in which most portions of light are transmitted without obvious reflection. However, they can still be detected by AFM, showing a terraced height difference of ~1.1 nm over the pristine area. The height difference can be attributed to the structural change after local oxidation.

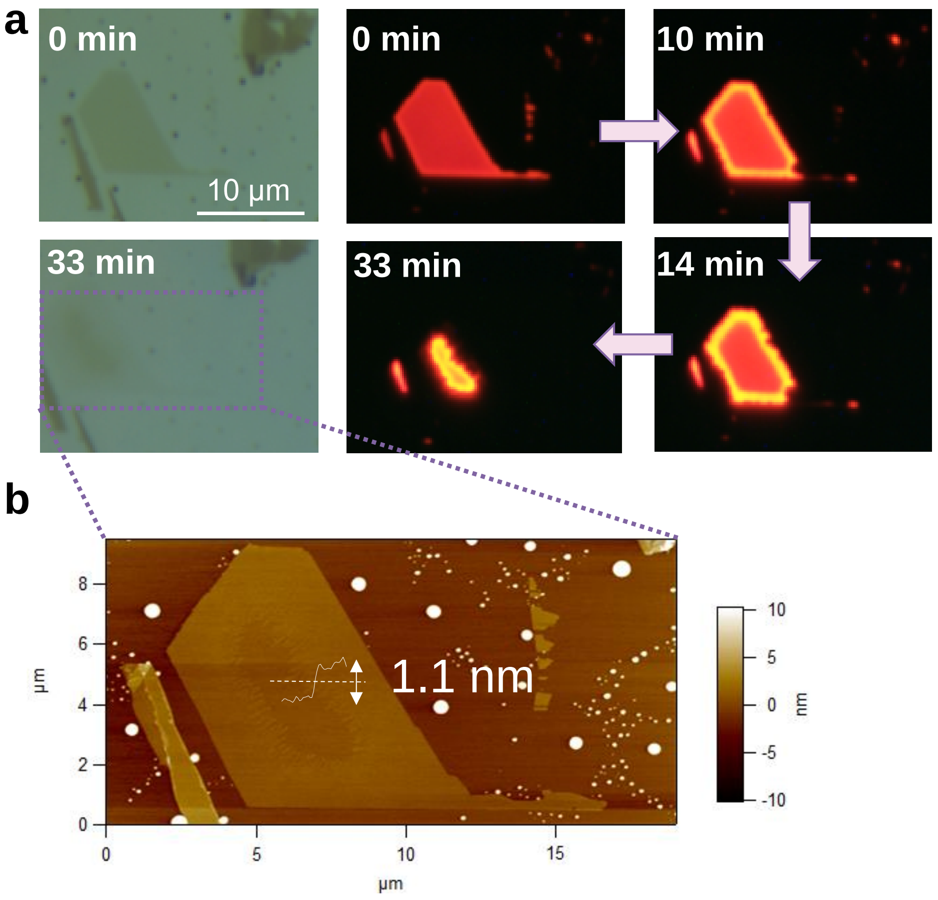

Supplementary Fig. 30 | Photo-oxidation activated via an alternative oxidant with TFSI molecules a, PL traced oxidation process of 1L WS_2 pretreated by TFSI molecules, by taking contrastive white-light reflection and PL images at different oxidation stages. **b**, Corresponding surface morphology by AFM for the sample after oxidation, where a height difference of 1.1 nm is recorded between the unoxidized and oxidized areas.

21. PL traced photooxidation for MoS_2

Supplementary Fig. 31a shows contrastive white-light reflection and serial PL images taken at different stages for a 30-min-pretreated, local 1L MoS_2 area with three edges surrounded by thick areas. Under continuous illumination with a focused 455 nm beam, the oxidation process initiates from all edges and develops as progressive frontal reaction

boundaries spreading inwards. We note that the sharp PL boundaries allow direct discrimination between the local pristine and oxidized regions for quantitative analyses, contrasting with previous Raman and AFM methods, in which the detected signals, i.e., the variations in Raman intensity or morphology, cannot distinguish between full and partial oxidation states.

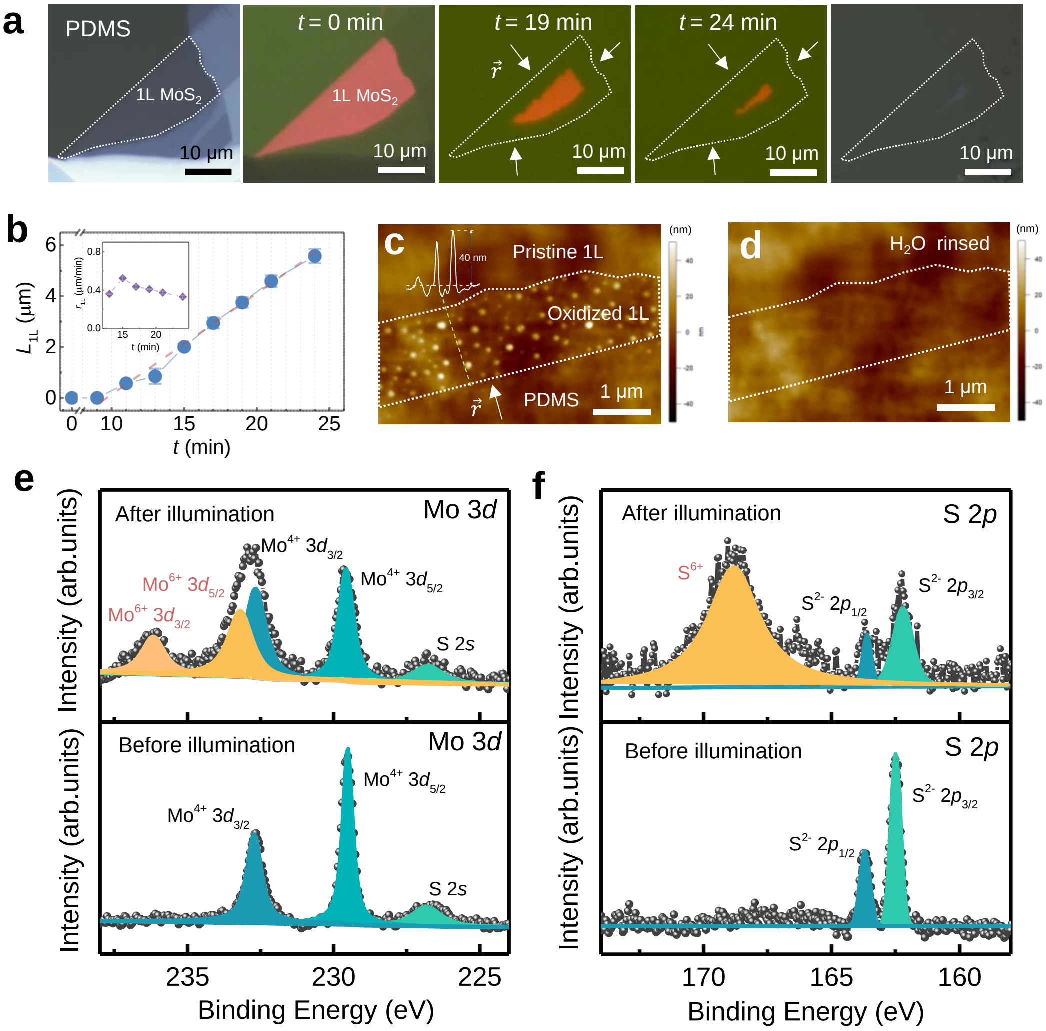

Supplementary Fig. 31 | PL traced photo-oxidation process in MoS_2. a, Contrastive white-light reflection and serial PL images taken at different stages for a 30-min pretreated 1L MoS_2. **b**, Oxidation length (L_{1L}) and rate (r_{1L}) versus illumination time (t). Standard deviations are used as the error bars. **c,d**, AFM images for the mixed pristine and oxidized local areas before and after rinse. **e**,**f**, Comparison of the characteristic (**e**) Mo 3*d* and (**f**) S 2*p* XPS modes before and after illumination. After oxidation, additional XPS modes (yellow shaded) arise from highly valent Mo and S atoms.

Supplementary Fig. 31b plots the oxidation length, L_{1L}, as a function of illumination time, t. In 24 min, L_{1L} reaches up to 6 μm and leads to a roughly constant etching rate, r_{1L}, of 0.4 ± 0.1 μm/min (inset of Supplementary Fig. 31b). To check the adhesion ability of the oxidization products, we also checked the surface morphologies before and after oxidization on a 3×5 μm^2 local area covering three regions: the pristine, oxidized, and PDMS substrate, as illustrated in Supplementary Fig. 31c,d. As can be seen, the small protrusions, with lateral sizes ranging from 20 to 100 nm, can be completely stripped after appropriate rinse in water, implying again their weak adhesive force to substrates. Such a clean strip of reaction residues constitutes a favorable merit for constructing clean and high-performance devices.

Analogous to WS_2, the oxidization products are complex oxysulfides and oxides showing reduced amount of S and highly valent Mo. By comparing the XPS spectra before and after oxidation, two W^{+6} excitation doublets emerge at 233 and 236 eV (Supplementary Fig. 31e). In addition, the intensities of S doublets are sharply reduced at 162.5 and 163.8 eV, as accompanied with a new mode around 169 eV. The behavior in XPS spectra is quite similar between MoS_2 and WS_2.

22. Statistics on [V] versus t_{pt} in MoS_2

Supplementary Figs. 33−37 shows typical atomically resolved STEM images for pristine and defect engineered MoS_2 samples for various pretreatment conditions. Supplementary Fig. 38 summarizes the raw statistical [V] distribution for different t_{pt} values. For each t_{pt} condition, we collected images from 25 independent 4×4 nm^2 areas in statistics. The values of average [V], A, and standard deviation, D, of the statistical data were obtained with Gaussian fittings. It was found that [V] increases linearly from 2.5 to 7.1×10^{13} cm^{-2} as t_{pt} increases from 0 to 30 min. The generation rate is estimated to be about 1.5×10^{12} cm^{-2} min^{-1} for 1L MoS_2 in 30% H_2O_2 solution, which is 5-fold lower than that of 1L WS_2, implying a slightly higher barrier for reaction between MoS_2 and H_2O_2.

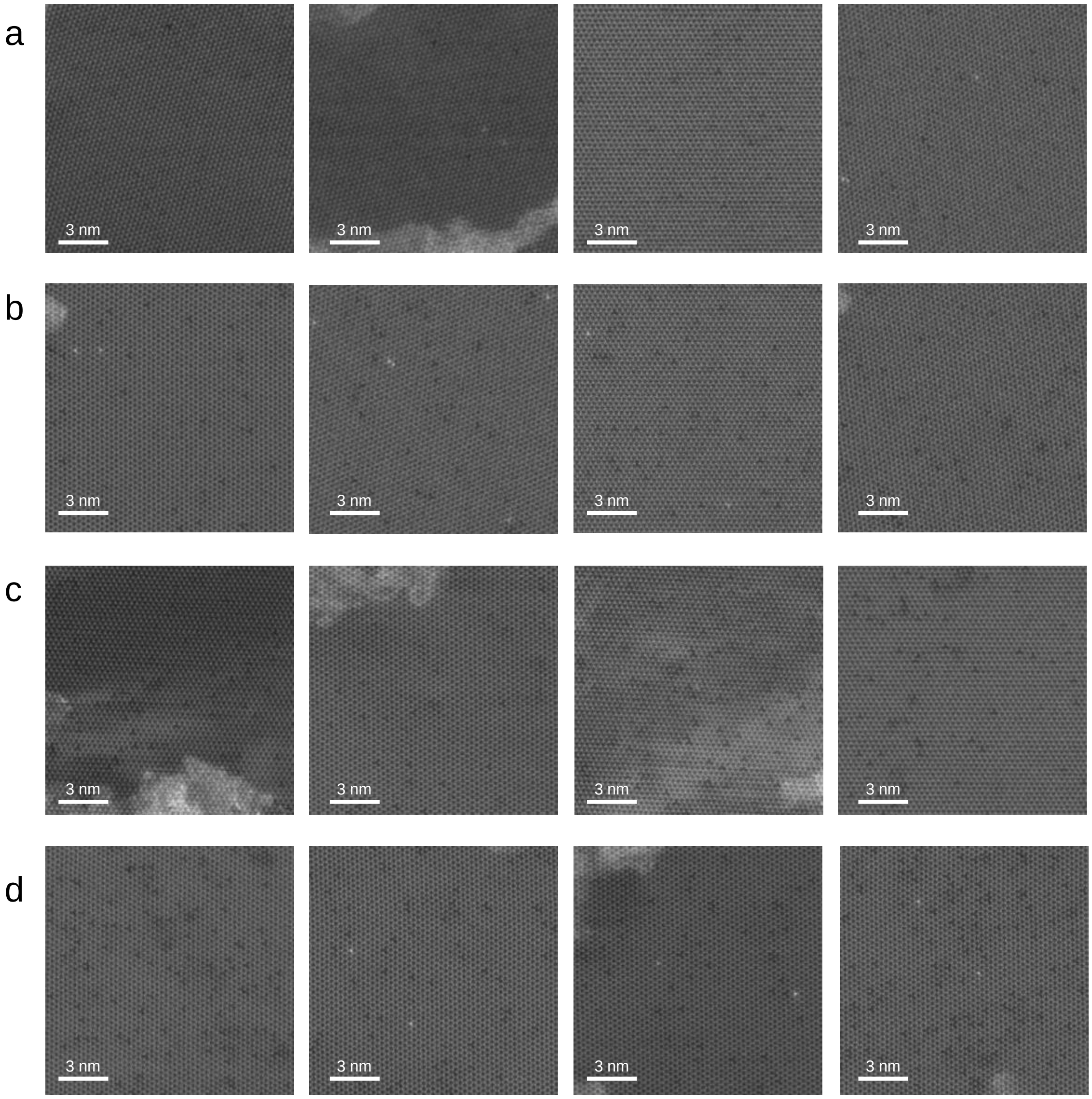

Supplementary Fig. 33 | Large-area (15×15 nm^2) raw STEM images for 1L MoS_2 after treating with H_2O_2 solution for different durations: **a**, 0 min; **b**, 10 min; **c**, 20 min; **d**, 30 min.

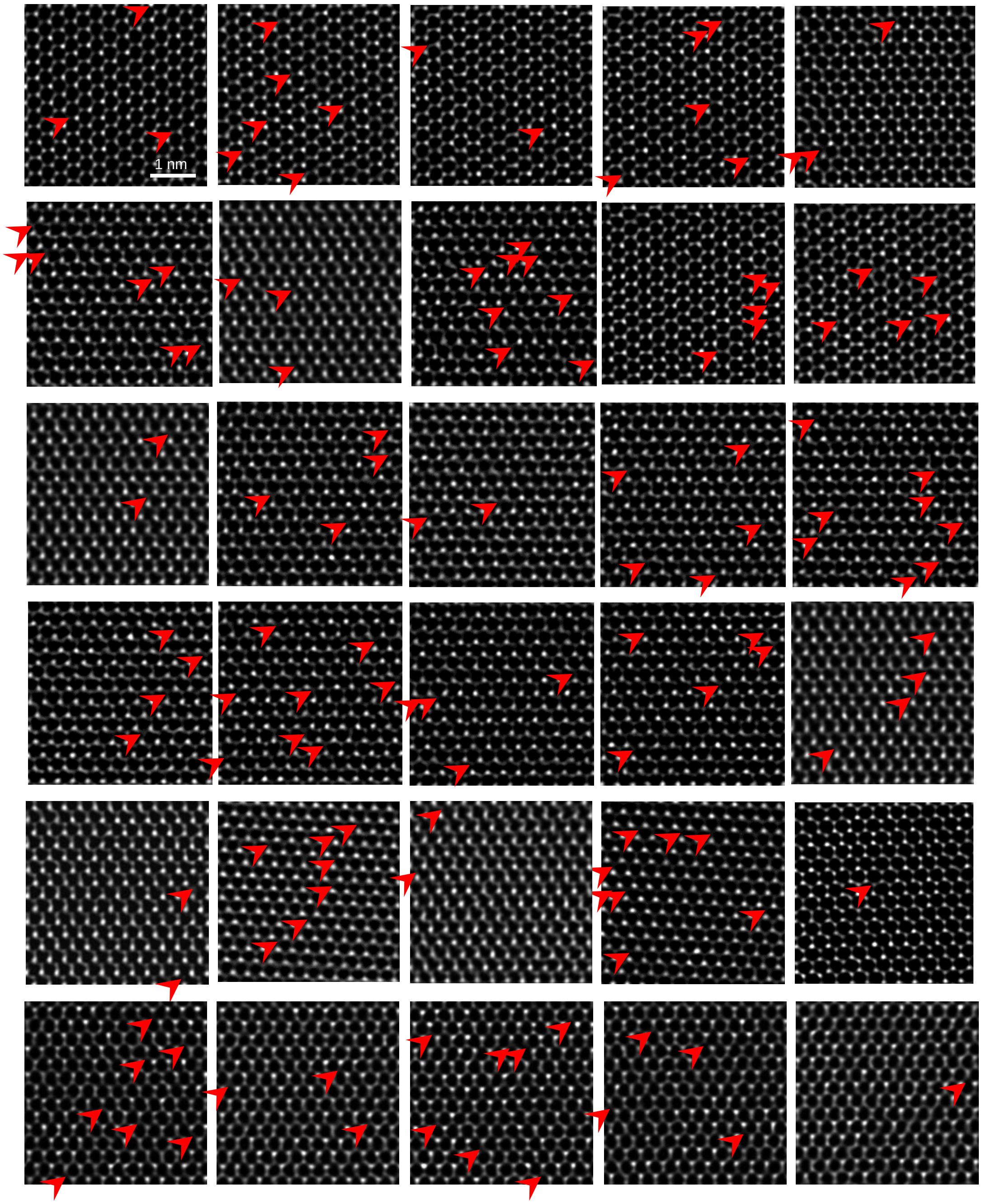

Supplementary Fig. 34 | Statistics of sulfur vacancy distribution in pristine monolayer MoS_2 without pretreatment. Scale bar: 1 nm.

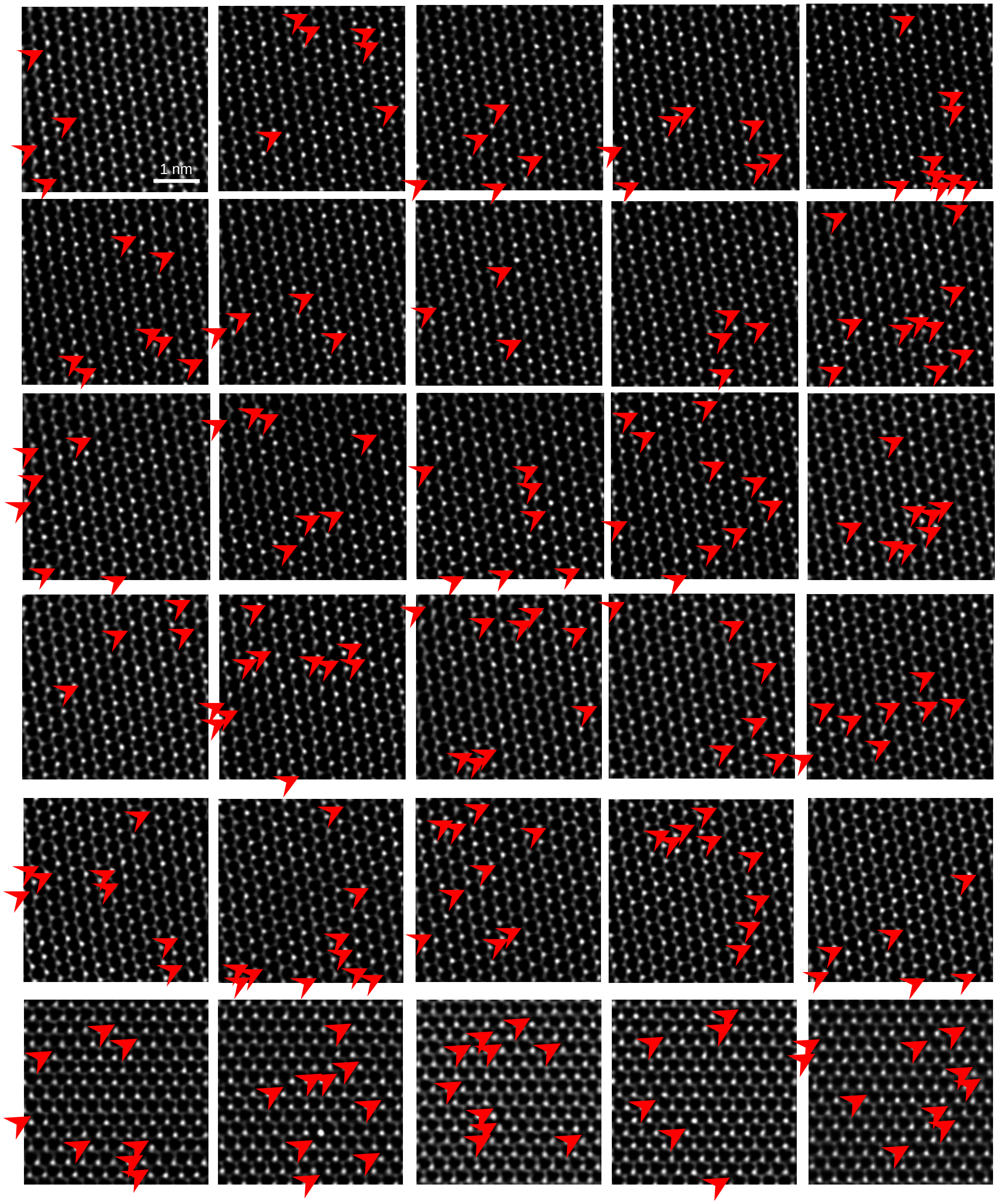

Supplementary Fig. 35 | Statistics of sulfur vacancy distribution in monolayer MoS_2 after pretreatment in H_2O_2 solution for 10 min. Scale bar: 1 nm.

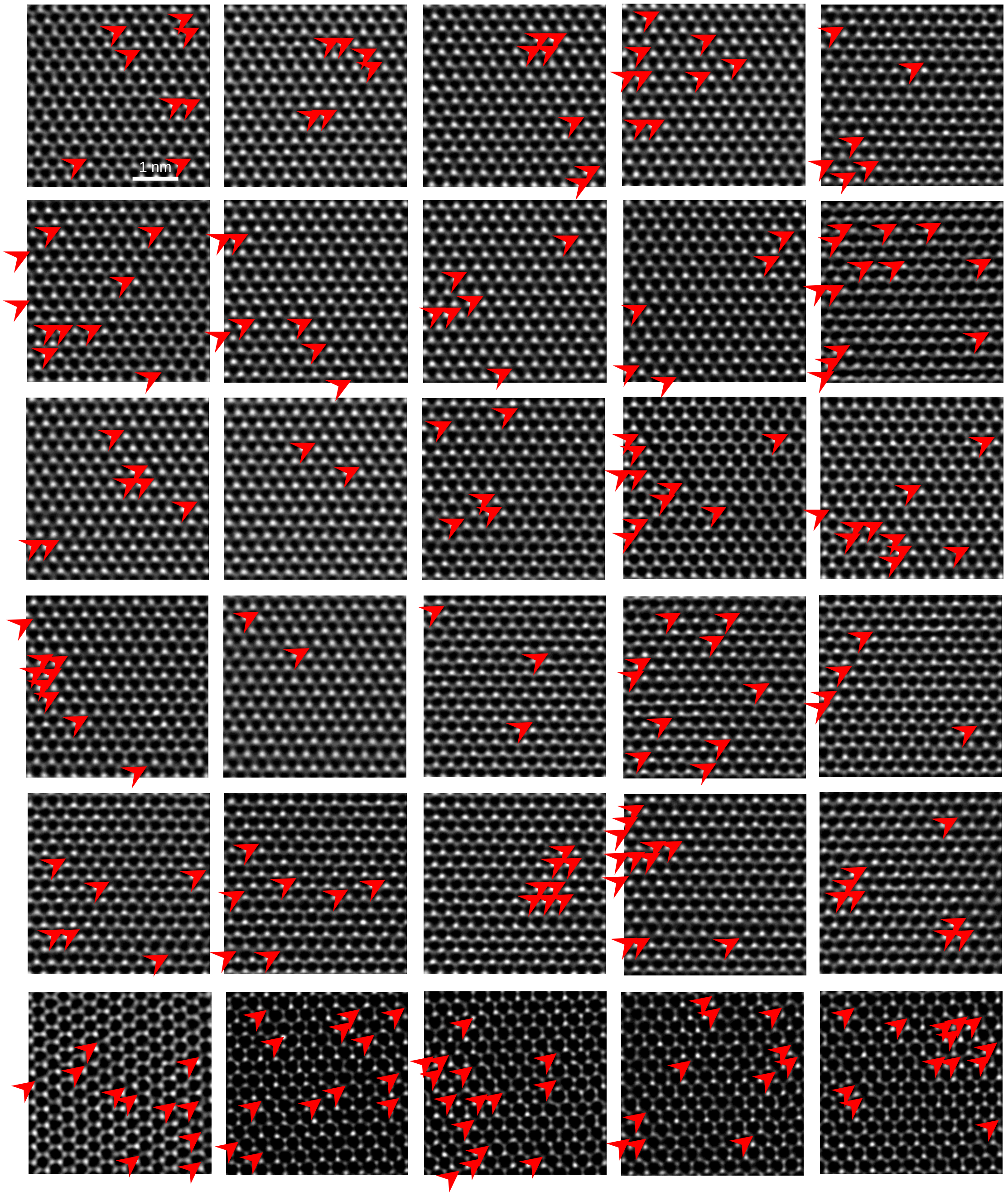

Supplementary Fig. 36 | Statistics of sulfur vacancy distribution in monolayer MoS_2 after pretreatment in H_2O_2 solution for 20 min. Scale bar: 1 nm.

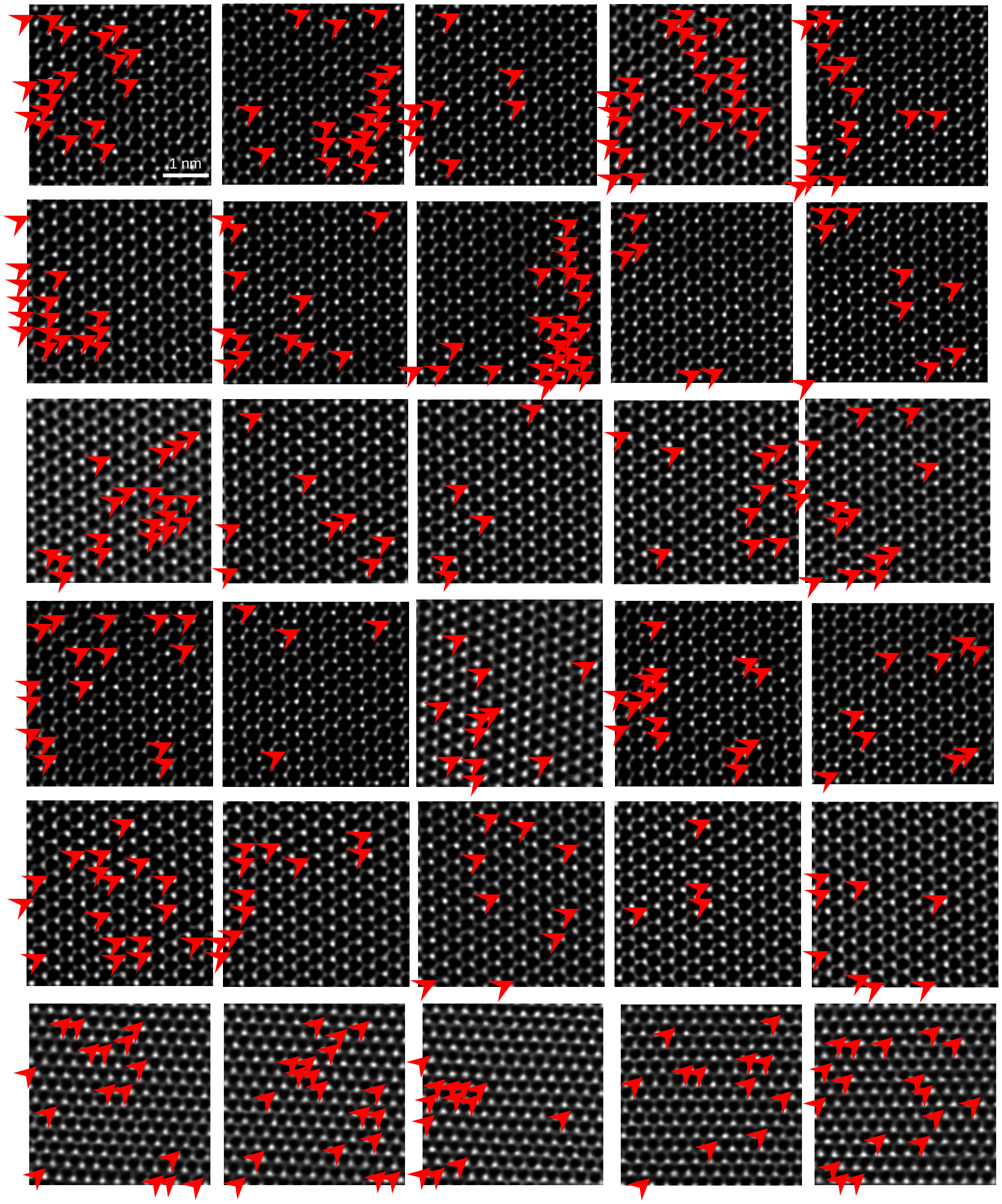

Supplementary Fig. 37 | Statistics of sulfur vacancy distribution in monolayer MoS_2 after pretreatment in H_2O_2 solution for 30 min. Scale bar: 1 nm.

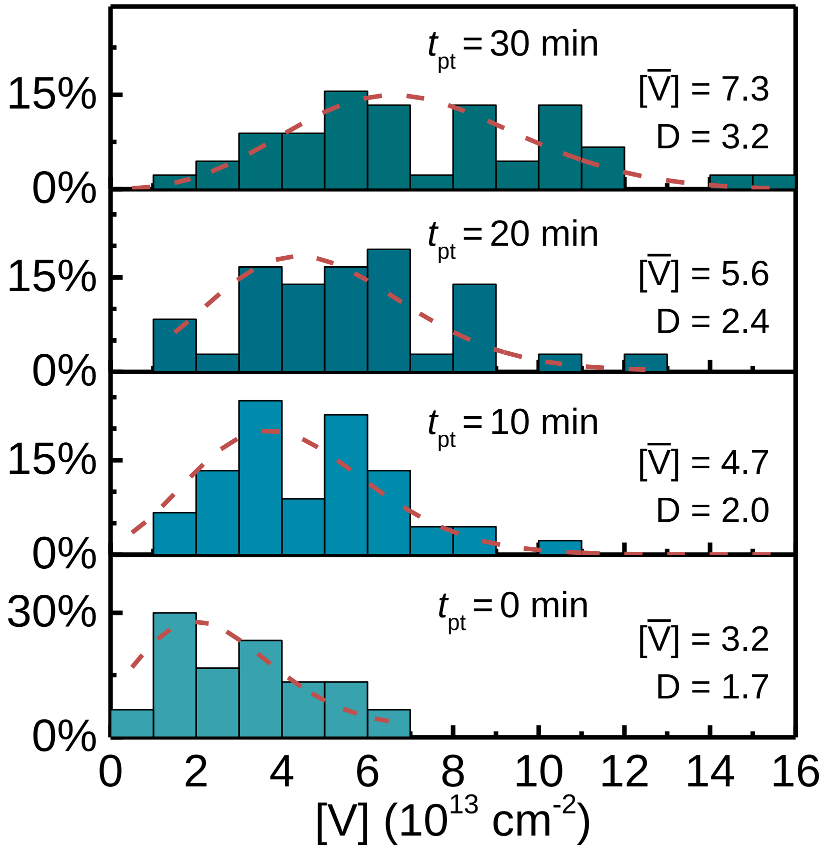

Supplementary Fig. 38 | Statistics on the vacancy distribution in 1L MoS_2 pretreated with dilute H_2O_2 solutions for different t_{pt} values. For each t_{pt}, more than 30 regions of an area of 4×4 nm^2 are used in the statistics. D denotes the standard deviation.

Supplementary References

1. Hong, J. *et al.* Exploring atomic defects in molybdenum disulphide monolayers. *Nat. Commun.* **6**, 6293 (2015).
2. Lin, Y.-C., Dumcenco, D. O., Huang, Y.-S. & Suenaga, K. Atomic mechanism of the semiconducting-to-metallic phase transition in single-layered MoS_2. *Nat. Nanotechnol.* **9**, 391–6 (2014).
3. Wei, J. *et al.* Enhanced thermal conductivity of polydimethylsiloxane composites with carbon fiber. *Compos. Commun.* **17**, 141–146 (2020).
4. Hu, L., Shan, X., Wu, Y., Zhao, J. & Lu, X. Laser thinning and patterning of MoS_2 with layer-by-layer precision. *Sci. Rep.* **7**, 15538 (2017).
5. Favron, A. *et al.* Photooxidation and quantum confinement effects in exfoliated black phosphorus. *Nat. Mater.* **14**, 826–832 (2015).
6. Kang, J., Tongay, S., Zhou, J., Li, J. & Wu, J. Band offsets and heterostructures of two-dimensional semiconductors. *Appl. Phys. Lett.* **102**, 012111 (2013).
7. Li, S.-L. *et al.* Nano-subsidence-assisted precise integration of patterned two-dimensional materials for high-performance photodetector arrays. *ACS Nano* **13**, 2654–2662 (2019).
8. Amani, M. *et al.* Near-unity photoluminescence quantum yield in MoS_2. *Science* **350**, 1065–1068 (2015).
