# Supplementary material for: Oxidation kinetics and non-Marcusian charge transfer in dimensionally confined semiconductors
Source: Nat Commun. 2023 Jul 10;14:4074. doi: 10.1038/s41467-023-39781-y (PMC10333350; doi:10.1038/s41467-023-39781-y)
Supplement: Supplementary file 2 — Description of Additional Supplementary Files [file 41467_2023_39781_MOESM2_ESM.pdf]

Description of Additional Supplementary Files

File Name: Supplementary Movie 1

Description: Photooxidation process for 1L WS_2 traced under photoluminescent imaging.

File Name: Supplementary Movie 2

Description: Photooxidation process for 1L MoS_2 traced under photoluminescent imaging.
